# Biodistribution and Toxicological Impact Assessment of Cerium Dioxide Nanoparticles in Murine Models: A Systematic Review of In Vivo and Ex Vivo Studies

**DOI:** 10.3390/pharmaceutics17111475

**Published:** 2025-11-16

**Authors:** Polina I. Lazareva, Victor A. Stupin, Kirill A. Lazarev, Petr F. Litvitskiy, Natalia E. Manturova, Ekaterina V. Silina

**Affiliations:** 1 I.M. Sechenov First Moscow State Medical University (Sechenov University), 119991 Moscow, Russia; p.dubrovina@mail.ru (P.I.L.);; 2 Pirogov Russian National Research Medical University, 117997 Moscow, Russia; stvictor@bk.ru (V.A.S.);

**Keywords:** cerium oxide, nanoparticles, nanoceria, biodistribution, toxicity, accumulation, pharmacokinetics, in vivo, ex vivo, mice, animals

## Abstract

**Background/Objectives:** This review summarizes and analyzes current data on the toxicological effects of cerium dioxide nanoparticles (nanoceria) on various anatomical and functional systems in healthy murine models, as reported in both in vivo and ex vivo experimental settings. **Methods:** This systematic review was conducted and reported in accordance with the PRISMA 2020 guidelines and was prospectively registered in PROSPERO (CRD42024503240). A systematic literature search was conducted using the PubMed and ScienceDirect databases for the period 2019–2025, with the inclusion of earlier publications having significant scientific relevance. The final search update was conducted in July 2025 to ensure inclusion of the most recent studies. **Results and Conclusions:** Only in vivo and ex vivo studies in healthy murine models were included. Risk of bias was evaluated using the OHAT tool for animal studies, and data were synthesized narratively due to heterogeneity among studies. A total of 29 studies met the inclusion criteria. The pharmacokinetic properties of nanoceria were considered, encompassing biodistribution, elimination pathways (including oral, intravenous, intraperitoneal, inhalation, intratracheal, and instillation routes), and the influence of physicochemical characteristics on bioavailability and toxicity. The toxicological impact (TI) was assessed across major organ systems—respiratory, digestive, urinary, visual, reproductive, nervous, cardiovascular, immune, hematopoietic, endocrine, musculoskeletal, and skin. The liver, spleen, lungs, and kidneys were identified as primary accumulation sites, with clearance dependent on particle size and coating. The TI spectrum ranged from the absence of morphological changes to inflammation, fibrosis, or organ dysfunction, depending on dose, exposure route, and physicochemical parameters. The main limitations include variability of nanoparticle formulations and incomplete toxicity reporting. In general, CeO_2_ nanoparticles with sizes of 2–10 nm and doses ≤ 5 mg/kg showed no signs of systemic toxicity in short-term studies on healthy mice, provided that optimal coating and dosing intervals were used.

## 1. Introduction

The advancement of nanotechnology and its successful application in technical solutions [1,2,3,4,5,6] has generated significant interest among researchers working on its biomedical applications [7,8,9,10,11,12,13]. However, the enthusiasm of some researchers has been met with concerns from others, driven not only by the potential environmental toxicity of nanoparticles [14,15,16,17] but also by their ability to penetrate cells and disrupt the function of intracellular organelles and DNA itself [18,19,20,21,22].

In recent decades, rare earth metals (lanthanides) have been actively introduced into medical practice for their ability to enhance the regenerative properties of tissues. One such metal is cerium [23,24,25,26,27,28,29,30]. Its uniqueness lies in its redox activity: it can exhibit trivalent and tetravalent states [31]. The two primary oxides of cerium are cerium dioxide (CeO_2_) and cerium(III) oxide (Ce_2_O_3_). Both oxide states (+3 and +4) coexist on the surface of cerium nanoparticles. Cerium dioxide nanoparticles (CeO_2_ NPs, nanoceria) possess a set of unique physicochemical properties that determine their biological activity and distinguish them from many other engineered nanomaterials. Their crystal lattice is characterized by oxygen vacancies, which generate a high density of surface defects and confer remarkable redox activity. The ability of cerium to switch between the Ce^3+^ and Ce^4+^ oxidation states under physiological conditions allows nanoceria to mimic the activity of antioxidant enzymes such as superoxide dismutase, catalase, and peroxidase. This redox cycling is self-regenerating, enabling nanoceria to repeatedly participate in oxidative and reductive reactions without losing catalytic capacity. At the same time, under acidic or pathological microenvironments, nanoceria may exhibit pro-oxidant properties by promoting reactive oxygen species (ROS) production, which underlies their selective cytotoxicity against cancer cells [32,33,34,35,36]. The physicochemical profile of CeO_2_ NPs—including particle size, hydrodynamic diameter, shape, surface charge (ζ-potential), and type of surface coating—critically determines their stability in biological fluids, interaction with proteins and cells, and subsequent biodistribution and clearance. For example, particles <5–6 nm may undergo renal filtration, while larger or positively charged particles tend to persist longer in tissues or accumulate in the reticuloendothelial system. The formation of a protein corona further modifies their biological identity, influencing circulation time and the ability to cross barriers such as the blood–brain barrier [37,38].

Because of these properties, CeO_2_ NPs have attracted attention in a wide spectrum of biomedical applications. Preclinical studies have demonstrated their potential in neuroprotection (Parkinson’s disease, Alzheimer’s disease, ischemic stroke), ophthalmology (treatment of dry eye disease and retinal degenerations), cardiovascular protection, wound healing and tissue regeneration, mitigation of chronic inflammation (e.g., rheumatoid arthritis), radioprotection, and as adjuvant agents in cancer therapy due to their ROS-mediated cytotoxicity [39,40,41,42,43,44,45,46,47,48,49,50,51]. This versatility underscores the importance of understanding both their beneficial and toxicological effects in vivo.

In this review, the term “nanoceria” consistently refers to cerium oxide nanoparticles (mostly CeO_2_ NPs) containing both Ce^3+^ and Ce^4+^ surface oxidation states, as this dual redox activity is fundamental to their biological effects. In its bulk form, Ce^4+^ is more stable than Ce^3+^ due to its electron shell configuration, [Xe]4f^0^ versus [Xe]4f^1^ [52]. Therefore, the term “cerium oxide” in the literature most often refers to its dioxide, which is also denoted as ceria.

Despite the similar physicochemical properties of cerium and nanoceria, the latter has more surface defects associated with oxygen vacancies. This imparts autocatalytic properties and higher redox activity to nanoceria [53].

The Ce^3+^/Ce^4+^ ratio can be regulated by changing environmental conditions. For example, treating the surface of nanoceria with ascorbic acid or irradiating it with a green laser increases the proportion of Ce^3+^, while doping with europium has the opposite effect [54,55,56]. These examples were selected illustratively to represent three distinct approaches: chemical (ascorbic acid), physical (laser), and doping-based (europium). Other methods have also been described, but these three are among the most representative and well-studied. The biological behavior of nanoceria is governed by its oxidation state. Ce^3+^ confers antioxidant capabilities through its enzyme mimetic activity, replicating the functions of catalase, superoxide dismutase (SOD) [57], and peroxidase [58]. In contrast, Ce^4+^ demonstrates pro-oxidant and anti-tumor effects by infiltrating cancer cells and inducing cytotoxic reactive oxygen species (ROS) within the acidic tumor microenvironment [59].

At neutral pH, nanoceria exhibits cytoprotective properties, while in the acidic environment, it demonstrates pro-oxidant and cytotoxic effects [60]. An important property of nanoceria is its ability to repeatedly change its valence state by gaining or losing oxygen atoms without significant structural rearrangements, allowing for its reuse in redox reactions without loss of activity [61].

These properties make nanoceria a promising material for creating composites aimed at treating diseases associated with oxidative stress (OS), such as neurodegenerative diseases [62,63], diabetes [64], retinopathies [65,66], cardiovascular diseases [67], chronic inflammation (e.g., rheumatoid arthritis [68]), and tumor diseases [69,70,71,72], as well as for wound healing [73]. However, to date, despite its high potential value, nanoceria is far from therapeutic application and clinical trials due to an insufficient understanding of its toxicity to living organisms. At the same time, numerous studies have reported that cerium exerts different cytotoxic effects on cancerous and healthy mammalian cells [69]. This review pays special attention to studies investigating the toxicity of cerium in vivo and/or ex vivo in healthy murine models to predict the potential side effects of using nanoceria-based preparations.

At present, evidence regarding the effects of CeO_2_ nanoparticles in humans is extremely limited, with only isolated accidental exposure reported. Therefore, this review is restricted to murine in vivo studies in order to establish a baseline toxicological profile.

Thus, the rationale for this review lies in the fact that most existing publications have focused on individual organs, mechanisms, or in vitro models, whereas a systematic assessment of biodistribution and toxicological effects of CeO_2_ nanoparticles in healthy murine models has so far been lacking. Studies involving animals with pathological conditions, including tumor-bearing models, were excluded, since the objective of this review was to assess biodistribution and toxicity in healthy organisms in order to establish a baseline safety profile. Pathological models, while important for therapeutic research, may involve altered physiology and redox balance that would confound toxicological evaluation. Murine models were chosen because, at present, they represent a convenient, cost-effective, and rapid in vivo system for evaluating the safety of nanomaterials. The novelty of our review is its focus on preclinical experiments in healthy animals, which allows toxic effects to be evaluated without the confounding influence of pathology. The contribution of this work is to integrate scattered data, identify consistent patterns and limitations, and thereby provide a basis for future research planning and evaluation of clinical perspectives. This review also provides an opportunity to identify potential pitfalls and avoid difficulties when planning future research on the biological properties of CeO_2_ nanoparticles.

## 2. Materials and Methods

**Figure 1:**
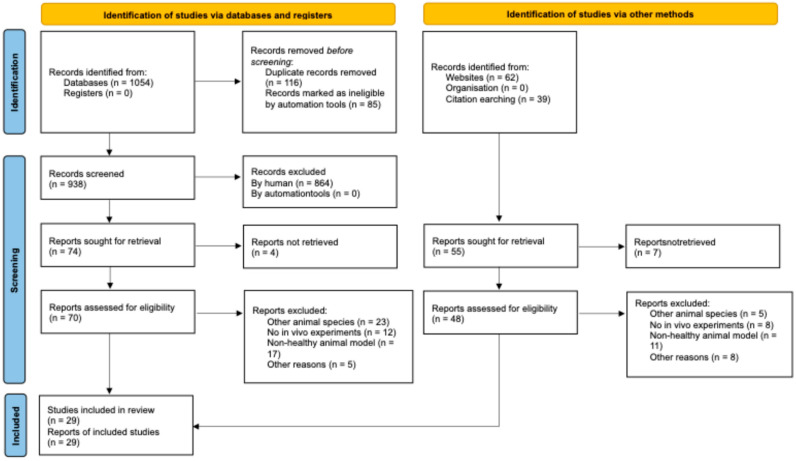
PRISMA 2020 flow diagram for new systematic reviews that included searches of databases, registers and other sources.

A literature search was conducted in the scientometric databases PubMed and ScienceDirect (Elsevier) to prepare this review. The process of identification, eligibility assessment, and inclusion of studies is summarized in Figure 1. Priority was given to articles published in English between 2019 and 2025. 

Although our primary inclusion criterion was publication between 2019 and 2025, we also incorporated a limited number of studies published before 2019. These earlier works were included if they met the following conditions: (i) publication in peer-reviewed journals with sufficient methodological quality; (ii) presentation of unique experimental data not reproduced in later studies (e.g., novel biodistribution pathways, organ-specific toxicity profiles, or rare administration routes); and (iii) provision of detailed experimental design and outcomes enabling reliable data extraction. This approach was adopted to ensure the completeness of the review and to avoid omission of pivotal findings that remain relevant to the current understanding of CeO_2_ nanoparticle biodistribution and toxicity.

The systematic review protocol was registered in the international PROSPERO database (International Prospective Register of Systematic Reviews; https://www.crd.york.ac.uk/prospero/ (accessed on 01 September 2025)) under the registration number CRD42024503240. Registration was completed prior to data collection and analysis. All methods and inclusion criteria were implemented as initially planned.

This systematic review was conducted following the PRISMA 2020 statement (Preferred Reporting Items for Systematic Reviews and Meta-Analyses).

The search was performed using the keywords nanoceria, cerium dioxide nanoparticle, and cytotoxicity and the search query (nanoceria OR “cerium oxide” OR “cerium dioxide nanoparticle”) AND (distribution OR accumulation OR pharmacokinetics OR disposition OR localization) AND (“in vivo” OR “ex vivo”) AND (mice OR mouse) AND cytotoxicity. A total of 1054 publications were found using this query. After assessing their relevance against the selection criteria and reading the full texts, 29 original studies were included in the review.

The search strategy was further refined with additional queries to analyze the TI on specific anatomical and functional systems. The inclusion criteria considered studies using in vivo or ex vivo murine models, employing nanoceria, and containing an evaluation of TI on specific anatomical and functional systems. The search employed Boolean operators and English keywords including “cerium dioxide” OR “CeO_2_ nanoparticles”, “toxicity OR toxicological impact”, mice OR mouse OR murine, “in vivo” OR “ex vivo”, and the names of the relevant organ systems. Publications focusing on the following systems were included: respiratory, digestive, urinary, visual, reproductive, nervous, cardiovascular, immune, endocrine, hematopoietic, musculoskeletal, integumentary (skin), and lymphatic. Furthermore, articles identified by their DOI, PMID, and other external links were considered, provided they were indexed in international scientific databases such as Web of Science, Scopus, SpringerLink, and Wiley Online Library.

During full-text screening, several articles that initially appeared relevant based on title and abstract were excluded after detailed assessment. The main reasons for exclusion included: (i) in vitro or non-murine models; (ii) studies involving cerium compounds other than CeO_2_ nanoparticles; (iii) lack of quantitative or descriptive toxicological outcomes; (iv) reviews, conference abstracts, or duplicated datasets; and (v) insufficient methodological details preventing reliable data extraction.

The extracted data items included study design, animal species and strain, nanoparticle characteristics (size, shape, coating, surface charge), administered dose and route, exposure duration, and all reported toxicological outcomes. The outcomes sought encompassed biodistribution, oxidative stress, histopathological changes, biochemical parameters, and organ-specific functional alterations. If multiple doses, time points, or experimental conditions were reported, all relevant results were extracted and analyzed to capture the full range of toxicological responses.

In addition to toxicological outcomes, data were collected on study design (in vivo or ex vivo), animal species, strain, sex, and age, as well as nanoparticle characteristics (size, morphology, coating type, surface charge, and administered dose). Information on the route of administration, exposure duration, and study funding (if reported) was also extracted. When details were missing or unclear, the information was recorded as “not specified” without assumptions or imputation.

The review included studies investigating both pure cerium dioxide and some composite materials based on it, provided they retained the key pharmacokinetic properties of cerium dioxide, or their comparison with “pure” nanoceria was of interest for the analysis.

Prior to synthesis, all extracted data were reviewed for consistency of units and reporting formats. Doses were standardized to mg/kg body weight, and nanoparticle sizes were expressed in nanometers. When studies presented data qualitatively (e.g., “no significant change” or “increase”), these findings were categorized descriptively. Missing or unclear information was reported as “not specified” without imputation or estimation. No data conversions or statistical imputations were performed. Results from individual studies were tabulated by organ system and experimental parameters (nanoparticle characteristics, dose, route, and duration of exposure). Data were visually summarized using descriptive tables to facilitate comparison across studies.

Additional article selection criteria included the structured nature and logical flow of the presentation, the relevance and reliability of the presented data (including references to primary sources), the relevance of the authors’ scientific publications to the topic, and the citation count of the work.

Literature reviews, systematic reviews, studies on in vitro cell cultures, studies on animals other than mice, and the presence of severe comorbidities (including oncological diseases) in the animal models were excluded.

Certainty (confidence) in the body of evidence was not quantitatively assessed (e.g., using GRADE) because the studies were preclinical and highly heterogeneous in methodology. The overall strength of evidence was therefore evaluated qualitatively based on study quality and consistency of findings.

Studies meeting the inclusion criteria were organized according to their experimental design, exposure route, and primary outcome domain. To ensure consistency across syntheses, individual studies were tabulated by organ system (e.g., hepatic, pulmonary, renal, neural, reproductive, and others) and compared in terms of nanoparticle characteristics (size, coating, dose, and duration of exposure). This approach allowed identification of common trends in biodistribution and TI across comparable experimental conditions.

Two reviewers independently screened titles and abstracts, extracted data, and assessed study eligibility. Any disagreements were resolved through discussion.Risk of bias was assessed using the OHAT tool for animal studies.

The risk of bias for animal studies was evaluated using the OHAT Risk of Bias Rating Tool for Human and Animal Studies. This tool was selected because it is specifically designed for toxicological and experimental animal research, allowing assessment of internal validity across domains such as exposure characterization, outcome assessment, and reporting. The OHAT approach was considered more appropriate for this review than SYRCLE’s tool, which is limited to experimental design features and does not cover exposure or analytical bias relevant to nanotoxicology.

No quantitative effect measures (e.g., risk ratio, mean difference) were calculated due to the heterogeneity of study designs, endpoints, and measurement methods. The findings were summarized narratively and presented in descriptive tables according to organ system and exposure parameters. In addition, due to the heterogeneity in experimental design, animal species, and nanoparticle formulations, no subgroup or sensitivity analyses were performed. Variations among studies were discussed qualitatively in terms of model type, dose range, and physicochemical characteristics of nanoceria.

The review did not include a formal statistical evaluation of reporting bias or small-study effects, as no meta-analytic pooling was performed. However, potential bias related to publication and selective outcome reporting was considered in the Discussion and Limitations sections. No formal sensitivity analysis was conducted because a quantitative synthesis was not performed. However, consistency of the findings was evaluated qualitatively by comparing studies across different experimental models, doses, and nanoparticle coatings, which confirmed the robustness of the overall conclusions. No formal statistical assessment of reporting bias was conducted because a quantitative synthesis was not performed. However, the potential risk of bias arising from selective reporting or publication of predominantly positive findings was considered qualitatively. Several included studies lacked full disclosure of negative or null results, which may have led to a modest overestimation of nanoceria safety in preclinical models.

## 3. Results

### 3.1. Pharmacokinetics and Pharmacodynamics of Nanoceria

Despite active research into the antioxidant, anti-tumor, and regenerative properties of nanoceria, their pharmacokinetic behavior in the body remains poorly understood. How these nanoparticles distribute within tissues, how long they persist in the body, their elimination routes, and the degree to which these parameters depend on the particles’ physical characteristics (size, coating, charge, hydrophobicity, etc.). All of this directly affects their efficacy and safety as potential therapeutic agents.

This section reviews 12 original studies that investigated various aspects of the pharmacokinetics and pharmacodynamics of nanoceria in vivo and ex vivo (Table 1). This review comprehensively examines classical research on nanoparticle biodistribution and recent works addressing its elimination, coating- and charge-dependent biological behavior, and antioxidant–toxicological properties. The studies are grouped in a logical sequence: from basic models of biodistribution and elimination to research focusing on the influence of stabilizers, protein coronas, immune response, and route of administration on the biological properties of the nanoparticles. This approach allows for a comprehensive examination of nanoceria behavior in the body and identifies key parameters determining their safety and efficacy during systemic application.

The risk of bias for the included in vivo and ex vivo studies was assessed using the OHAT Risk of Bias Rating Tool (2015). Overall, the studies demonstrated low to probably low risk of bias. Most experiments were conducted under institutional ethical approval and in compliance with OECD or GLP standards, with clearly defined and parallel control groups, which reduces the likelihood of selection and performance bias. Several studies explicitly described random assignment or blinded assessment of outcomes, while others provided sufficient methodological detail to infer that animals were treated under uniform and unbiased conditions. No indications of attrition, selective reporting, or other systematic errors were identified. Consequently, the overall internal validity of the included animal studies can be considered high, supporting the reliability of the experimental results.

The first study in this list investigated the pharmacokinetics of nanoceria following their intravenous administration at low doses [74]. C57BL/6 mice were injected with nanoparticles at doses of 0.1 and 0.5 mg/kg through the tail vein. Some animals were sacrificed on day 7 of the experiment, while the remaining mice received a second administration of nanoceria on day 15 and were sacrificed on day 30. Histological analysis and transmission electron microscopy (TEM) showed that nanoceria persists over time in the tissues of the liver and kidneys and within the lumen of the tail vein vessels. Specifically, electron-dense granules sized 100–200 nm were localized in the cytoplasm of hepatocytes, the epithelium of renal tubules, and the plasma of tail vein blood vessels. Repeated nanoceria dosing (day 15) and a prolonged observation period (30 days) revealed no inflammatory, necrotic, or pathological changes in the examined organs (liver, kidneys, lungs, brain, pancreas, spleen), demonstrating its favorable biocompatibility. However, the obtained data have several limitations. These include the lack of quantitative data on accumulated cerium dioxide: the granules visualized by TEM are highly likely to be nanoceria, but this was not confirmed analytically. The use of, for example, inductively coupled plasma mass spectrometry (ICP-MS) could have identified the granules and analyzed their dynamics: whether nanoceria accumulates in tissues over time or is gradually eliminated. Furthermore, the authors did not assess the routes of nanoparticle elimination, which prevents a complete picture of their metabolism. The small sample size (only 4 mice from each group on days 7 and 30 of the experiment) is also a drawback and reduces the statistical power of the results. Nevertheless, the obtained data demonstrate the ability of nanoceria to persist in tissues for extended periods without triggering an acute inflammatory response, suggesting their potential application in treating chronic inflammatory conditions.

The next study is a comprehensive in vivo investigation comprising multiple experiments, including an assessment of pharmacokinetics via different administration routes, fluorescence imaging, and analysis of the antioxidant activity of nanoceria. It provides a holistic understanding of nanoceria behavior in the body over varying therapy durations. The goal of the first experiment on 36 CD-1 mice, administered nanoceria (0.5 mg/kg, diluted in 100 µL of saline), was to study the dynamics of cerium distribution throughout the body depending on the administration route: intravenous, oral, or intraperitoneal [74]. Mice were divided into groups based on the administration route and duration (once a week for either 2 or 5 weeks). According to the results, the highest deposition of nanoceria in tissues occurred after intravenous administration. Intraperitoneal and oral routes followed in terms of accumulation extent, respectively. In mice subjected to intravenous and intraperitoneal administration of cerium, the highest concentration of nanoceria (per gram of tissue) was found in the spleen, followed by the liver. Accumulation of nanoceria was also observed in the lungs and kidneys, but at lower concentrations. Regarding the heart and brain, no significant cerium accumulation was detected after oral administration; however, low concentrations of nanoceria were found after intravenous and intraperitoneal routes. In mice receiving nanoceria orally, organ deposits were negligible, except for low concentrations found in the lungs, which were most likely caused by the tracheal gavage procedure or minor aspiration. The elimination of cerium was studied through the kidneys and intestines. It was established that the primary route of nanoceria excretion was via feces. It was not detected in the urine of any group. These results are consistent across both the two-week and five-week observation periods.

It is noteworthy that the total nanoceria concentration (µg CNP/g of organ tissue) in the mice from the five-week study group (which received three additional cerium injections) was three times higher than in the two-week group. This suggests that cerium was scarcely eliminated from the studied organs during the experiment. Among the complications, splenic damage (unspecified) was found in one mouse with intraperitoneal cerium administration, an active response of the Peyer’s patches in the small intestine of another mouse, and pulmonary bleeding in two more mice that received intravenous cerium. All other side effects were minor and not considered significant. The detection of cerium in parenchymal organs after IV administration indicates its ability to penetrate endothelial cells. It is hypothesized that larger nanoceria sizes coupled with site-specific administration could reduce toxicity while amplifying therapeutic outcomes (although the exact sizes of the nanoceria used in this work were not specified). However, this assertion requires further investigation.

Blood parameters were also analyzed in these mice. Compared to the control group, mice treated with nanoceria showed minimal differences after two weeks of exposure. However, a tendency towards mild leukocytosis (up to 10 × 10^9^/L) was observed in the test subjects during the five-week study.

A significant addition to the study was the use of fluorescently labeled nanoceria, which was administered intravenously (via the tail vein) to another group of CD-1 mice (6 received nanoceria and 6 were in the control group) at a dose of 2.5 mg/kg. Fluorescence imaging was performed 3 h and 2 weeks after the injection. Analysis of the fluorescent images at the 3 h mark detected cerium not only in parenchymal organs (liver, spleen, and lungs) but also in small peripheral areas around the neck and axilla, possibly related to lymph node activity. Two weeks post-injection, nanoceria was still detectable in the tissues. This, along with the previous experiment, demonstrated that cerium could be suitable for long-term treatment courses (with particularly effective accumulation observed after intravenous administration).

As part of the same study, an analysis of the antioxidant activity of nanoceria was conducted on three groups, each comprising 10 mice. During the first week, subjects received administrations of lead sulfide, nanoceria (IV; 0.5 mg/kg), or N-acetylcysteine. In the following week, each group was subdivided, with subgroups receiving a repeated exposure to either lead sulfide or carbon tetrachloride as OS inducers. Blood samples (taken 3 h post-injection) and urine samples (taken 12 h post-injection) were also collected. In the third week, the pro-oxidant substance was re-administered according to the group assignment, and blood and urine samples were taken again. Malondialdehyde (MDA) from blood plasma and 8-OH-deoxyguanosine from urine were used as markers of OS (lipid peroxidation). According to the urine results in the second week, there were no significant differences in the concentration of the substance. In the third week, the results showed a slight decrease in the level of 8-OH-dG in all mice that had previously received cerium (with or without induced OS). Although the reduction in OS from nanoceria administration was not statistically significant, after 3 weeks, the measured parameters showed that nanoceria produced results similar to those of N-acetylcysteine in reducing OS induced by CCl_4_. Regarding malondialdehyde, a reduction in lipid peroxidation indicators was observed in mice that received cerium at 2 weeks, but it was statistically insignificant, unlike at week 3. N-acetylcysteine also acted as an antioxidant but was less effective than nanoceria. Interestingly, an increase in malondialdehyde levels was observed in the groups that received N-acetylcysteine and cerium dioxide without prior induction of OS [74].

This experiment demonstrated that nanoceria reduces ROS levels in vitro, but according to the in vivo results, the ability of nanoceria to absorb ROS can be influenced by other factors, which may either hinder the reduction in OS levels or induce a pro-oxidant effect of nanoceria.

The next study aimed to identify differences in the bioavailability and toxicity of nanoceria in mice of different genetic strains. The influence of the mouse strain on the uptake of nanoceria by organs and cells, and on cellular and OS, was studied ex vivo [75]. Two groups of mice, C57BL/6 and BALB/c strains (prone to Th1 and Th2 immune responses, respectively), were used, with 40 mice in each group. In each group, 30 mice were administered 10 mg/kg of nanoceria (particle diameter 4.2 ± 1.2 nm) intraperitoneally, and 10 mice served as controls. The mice were euthanized at 30 min, 1, 3, 6, or 24 h post-injection, and tissue samples were taken from the brain, heart, lung, kidney, spleen, and liver. As in the previous study, the highest concentrations of nanoceria were found in the liver and spleen (though in this study, it was higher in the liver, whereas in the previous one, it was higher in the spleen). The percentage of the administered dose found in the liver, spleen, kidneys, lungs, heart, and brain (for both strains combined) was 12%, 1.6%, 0.78%, 0.17%, 0.03%, and 0.003%, respectively. Over time, there was a general decrease in cerium in the heart and a significant decrease in the brain (compared to the heart) and an increase in the kidneys, liver, and spleen. The decrease in brain tissue cerium 30 min after administration is likely related to a decrease in blood cerium levels and its inability to cross the blood–brain barrier (BBB) [76]. Overall, more cerium accumulated in C57BL/6 mice than in BALB/c mice. The transformation of cerium in the liver (within phagolysosomes) and spleen occurred with the formation of cerium phosphate (CePO_4_) nanoneedles; an increase in ferritin levels was also observed in these areas. The higher level of caspase-1 in BALB/c mice compared to C57BL/6 mice is consistent with the reported susceptibility of M2-like cells compared to M1-like cells to nanoparticle exposure. C57BL/6 mice also exhibited increased vacuolization of liver cells (an adaptive response aimed at limiting damage), while BALB/c mice showed reduced vacuolization and an increase in the density of the splenic white pulp (a sign of immune system activation). The reduction in vacuolization is presumably associated with the protective (anti-inflammatory/anti-OS) effect of nanoceria. All this indicates a greater susceptibility of BALB/c mice compared to C57BL/6 mice to the effects of nanoceria, as C57BL/6 macrophages absorb cerium more effectively, thereby potentially mitigating its effects (both intended and side). These data underscore the importance of considering genetic differences in immune response when assessing the safety of cerium nanoparticles.

One of the studies was dedicated to the long-term observation of nanoceria distribution, confirmed by fluorescence imaging data [77]. The work also included histological and laboratory assessments of nanoceria toxicity upon systemic administration. This was an in vivo experiment on Swiss mice that received a single intravenous injection of nanoceria-based biopolymers at a dose of 5 mg/kg (size of uncoated particles: 9 nm). The control group received phosphate-buffered saline (PBS). The distribution of nanoceria was assessed using fluorescent ultrasound imaging from the first minutes after administration and for up to 28 days. Twenty-four hours after administration, nanoceria particles were detected in the liver (33.2 ± 1.6 µg/g) and spleen (29.8 ± 1.4 µg/g), with small amounts in the kidneys (3.3 ± 0.2 µg/g) and lungs (3.1 ± 0.2 µg/g). One month after injection, particles were still detected primarily in the spleen (10.1 ± 1.4 µg/g) and liver (3.2 ± 0.6 µg/g). A fluorescence peak was observed in the bladder area 30 min after administration, which rapidly decreased to negligible concentrations 6 h post-injection. However, cerium itself was not analytically measured in urine, and the transient fluorescence peak observed in the bladder was attributed to the Cy5 dye rather than to confirmed urinary excretion of nanoceria. It can be assumed that the fluorescence was caused by the filtration of cerium through the kidneys, but according to available data, the optimal size for renal clearance is nanoparticles no larger than 8 nm (particles up to 6 nm are freely filtered; filtration of particles 6-8 nm depends on charge interactions with the renal basement membrane [78]). In this study, the nanoceria size was 9 nm. It is possible that the fluorescence peak is not related to nanoceria themselves but to their metabolites or associated molecules (e.g., the fluorescent dye used in the study). This indicates the possible involvement of the kidneys in the elimination of nanoceria, despite their relatively large size. However, the lack of cerium detection in urine casts doubt on this conclusion and requires further investigation with more sensitive methods.

In these same mice, a histological examination of organ tissues (liver, spleen, kidneys, lungs, brain) and blood analysis were performed at 24 h and 1 month [77]. Regarding laboratory tests, although a slight relative lymphocytosis and leukocytosis were observed compared to the control group, all indicators were within the normal range. Regarding pathological examination, the nanoceria particles did not cause organ damage: no areas of fibrosis, necrosis, cellular regeneration, or immune or hemorrhagic infiltration were found. Minor damages were similar to those in the control group and were likely the result of post-mortem effects. The results of this study are consistent with previous experiments by other authors, leading to the conclusion that there is no pronounced toxicity of nanoceria (9 nm uncoated and 31.5 nm coated) at a dose of 5 mg/kg.

The next experiment included acute and subacute toxicological tests of high doses of nanoceria. The clinical behavior of the animals, biochemical markers, and morphological changes in organs were investigated. The goal of the acute test was to determine the lethal concentration and possible immediate TI after a single administration of nanoceria [79]. Three CD-1 mice were used, each receiving a different oral dose of nanoceria (2000, 3500, and 5000 mg/kg). The nanoceria sizes are not directly specified, but the authors reference another work where nanoceria of 5 nm size were synthesized [80]. During the 10-day observation period, all subjects survived, confirming the absence of acute lethal toxicity. No changes in mouse behavior were detected (the animals were active, showed no signs of stress or intoxication (tremors, lethargy, convulsions, reduced mobility, etc.), and consumed water and food as usual). There was no sharp weight loss in any of the mice, indicating that nanoceria did not cause serious systemic toxicity. However, inflammatory changes were identified: an increase in the number of leukocytes, creatinine, ALT, AST (three-fold), and bilirubin (seven-fold); histological analysis showed lymphocyte accumulation in the liver and kidneys. The biochemical and histological changes indicate that the kidneys and liver sustain the most significant damage from nanoceria exposure. Interestingly, at the dose of 5000 mg/kg, leukocyte levels were close to control levels, and liver enzymes increased only 1.2–1.5 times, likely due to nanoparticle aggregation at high concentrations and reduced bioavailability. Despite the importance of the experiment, the small sample size (one mouse per one nanoceria concentration) and the limited observation period (10 days) do not allow for definitive conclusions about the safety of nanoceria.

The subacute test assessed the maximum tolerated dose of nanoceria and its 14-day effects on mice. Despite high nanoceria doses (50/500/5000 mg/kg), all mice survived and did not experience significant weight loss (>10%). White blood cell and lymphocyte counts were elevated with low and medium doses but approached normal levels with the high dose, suggesting possible aggregation of nanoceria at high concentrations. Platelet and neutrophil levels were reduced by half, indicating potential bone marrow effects and immune response suppression. Similar to the acute test, increases in ALT, AST, creatinine, and bilirubin were observed. Histological analysis revealed lymphocyte infiltration in the liver and kidneys, as well as nanoceria agglomerates in alveolar macrophages of the lungs, suggesting potential particle dissemination via the bloodstream or aspiration during gavage. The stomach, intestine, spleen, ovaries, and testes showed no significant changes. Genotoxicity studies indicated minor DNA damage at the high nanoceria dose. The absence of inflammatory changes in the spleen was unusual, as prior studies show cerium oxide nanoparticles often deposit in this organ, warranting further investigation.

Subsequent work evaluated the systemic distribution and extrapulmonary effects of inhalational nanoceria exposure, highlighting their persistence in tissues and potential for delayed internal organ damage. Studies on inhalational exposure will be detailed in the “Respiratory System” section; regarding extrapulmonary effects, one experiment found nanoceria in the kidneys, liver, heart, and brain even 28 days post-exposure, indicating high biopersistence and slow clearance [81]. Blood count and biochemistry changes included neutrophilia, monocytosis, and lymphopenia from day 14 onwards. Although lymphocytes normalized during recovery, the persistent neutrophilic response suggests ongoing inflammation. Mice exposed for 28 days showed elevated urea and creatinine levels both during exposure and recovery, indicating increased renal load. Kidney histology revealed diffuse renal tubular degeneration, microvesicular cytoplasmic vacuoles in tubular cells, and tubular necrosis (on day 28 and during recovery). Urine contained cellular breakdown products, indicating renal tissue damage. Blood showed increased creatinine and urea levels (key markers of renal dysfunction). In the liver, nanoceria accumulated in Kupffer cells, intersinusoidal spaces, and around portal triads. Hepatocytes with multiple nuclei containing nanoceria were found in one mouse (day 28 post-exposure). ALT, AST, and bilirubin levels remained normal. Collectively, this suggests that while overt cytotoxicity might be absent in the liver, long-term nanoceria presence could lead to delayed organ changes. Heart histology found nanoceria in cardiac vascular endothelial cells, with some myocardial samples showing focal cytoplasmic vacuolization (degenerative changes). Although strong cardiotoxic changes were not recorded, nanoceria accumulation in cardiac endothelium could be a potential risk factor for vascular diseases. Nanoceria were detected in the brain only after 28 days of inhalation, and their levels remained stably high for the next 28 days post-exposure; however, no histopathological brain tissue changes were associated with nanoceria accumulation.

Another study demonstrated the influence of the stabilizer on nanoceria bioavailability and distribution, focusing on protein coronas and their role in particle penetration of the BBB. Nanoceria were studied as a therapeutic agent for multiple sclerosis, but healthy mice were used as the control group. The experiment showed that nanoceria accumulation depends on the stabilizer [82]. Stabilization with a citrate/ethylenediaminetetraacetic acid (CA/EDTA) mixture resulted in minimal accumulation in the liver and spleen (almost 100 times lower than non-stabilized or citrate-stabilized nanoceria), rapid clearance from these organs (most cleared within the first month, nearly absent by the fifth), and an inability to cross the BBB. In contrast, CA/EDTA-stabilized particles persisted in cerebellar, cortical, and hippocampal tissues for 5 months, although their concentration decreased significantly after 3–4 months. Repeated experiments yielded identical results [35]. This is likely due to nanoceria size (larger particles, 5–55 nm, aggregate in blood leading to macrophage uptake and rapid hepatic/splenic clearance), charge (−23.5 mV in this study, a moderately negative value compared to −55 mV for non-stabilized nanoceria, reducing immune recognition [37,38]), and crucially, according to the authors, differences in protein coronas [34,35,39]. The hard corona of non-stabilized nanoceria contains complement system proteins (C3, C4), fibrinogen, and immunoglobulins, causing rapid immune recognition and uptake by liver and spleen macrophages. The soft corona of CA/EDTA-stabilized nanoceria contains albumin and apolipoproteins (ApoA1, ApoE), which mask the nanoparticles from the immune system, allowing longer blood circulation, reducing macrophage uptake, and facilitating BBB penetration since ApoA1 and ApoE are involved in transporting lipophilic substances into the brain [83].

Another study investigated the effect of modifications with negatively charged polyacrylic acid (CeO_2_@PAA) and positively charged poly (2-(methacryloyloxy) ethyltrimethylammonium chloride) (CeO_2_@PMETAC) on nanoceria toxicity [84]. Female C57Bl/6 mice received intraperitoneal injections every 4–5 days (4 injections total) of nanoparticles at doses equivalent to 1.8, 5.3, and 16 mg Ce/kg body weight over 14 days. A comprehensive toxicological assessment was performed 24 h after the last injection (day 15). Only CeO_2_@PMETAC caused pronounced inflammatory changes, particularly in the capsules of the liver, spleen, and kidneys, and at the highest dose, with inflammation spreading into the liver parenchyma and leading to the development of necrosis and fibrosis. These animals also showed hematological deviations (neutrophilia, decreased MCH and MCV) and the appearance of megakaryocytes in the spleen (a sign of extramedullary hematopoiesis). CeO_2_@PMETAC also caused the greatest accumulation of nanoceria in the mononuclear phagocytic system organs (liver, spleen, bone marrow) compared to uncoated CeO_2_ and CeO_2_@PAA. None of the samples caused a significant increase in micronucleated erythrocytes or significant DNA damage according to the comet assay. The authors conclude that nanoparticle surface charge has a key influence on their toxicological properties in vivo.

In another study, which aimed in part to investigate nanoceria distribution, the authors showed differences in biodistribution, clearance rate, accumulation degree, and macrophage interaction depending on nanoparticle coating [85]. These results are important for selecting carriers for therapeutic purposes. For example, polyacrylic acid (PAA)-coated nanoceria were rapidly excreted renally, dextran–polyethylene glycol (DT10-PEG)-coated particles prolonged blood circulation for drug delivery, and DT10-NH_2_-coated particles enabled targeted liver macrophages, potentially useful for liver-related pathologies. Uncoated nanoceria accumulated instantly (1 min) in the lungs (due to aggregation) and subsequently in the liver and spleen and were slowly excreted (<3% over 7 days). Based on previous experimental results, long-term nanoceria exposure in an organ can cause dysfunction, making uncoated nanoceria unsuitable as therapeutic agents for internal use [61].

Another study examined the dependence of nanoceria excretion pathways on their size and coating type in the context of physicochemical properties. The authors evaluated the biodistribution of radiolabeled nanoceria in vivo and showed that 2 nm coated nanoparticles were excreted primarily via the renal route (blood clearance half-life ~10 min), while 6 nm particles were excreted via the hepatobiliary route (blood half-life ~27 min) [86]. The coating type also matters: this study used polyacrylic acid (PAA; particle size 2 nm) and dextran T10 (DT10; particle size 6 nm). PAA-nanoceria are smaller and negatively charged, leading to rapid renal excretion, while DT10-nanoceria are larger and have a lower charge compared to PAA, resulting in longer blood circulation and accumulation in the liver. Although the authors did not analyze urine for cerium content, indirect signs of biodistribution (rapid blood clearance, kidney accumulation, small size) indicate renal excretion of the nanoceria. Interestingly, the accumulation of nanoceria in organs varied depending on their size. For PAA-nanoceria, the kidneys were the primary accumulation organ; accumulation in other organs was temporary: in the liver and spleen within the first hour after administration, and in the lungs for several hours. For DT10-nanoceria, the main sites of accumulation were the liver (peak at 4 h, then decline) and the spleen (nanoceria accumulation increased over time), with early but insignificant accumulation also seen in the lungs and kidneys.

Another experiment suggested that the distribution of nanoceria depends on its characteristics and interactions with other cells. For instance, when captured by immune cells (particularly Kupffer cells), nanoparticles can remain within these cells until they degrade into ions (as cellular degradation occurs inside macrophages) and are subsequently eliminated via the urinary tract. In contrast, hepatocytes subject nanoceria to biliary excretion and, accordingly, hepatobiliary elimination [87]. Thus, if nanoceria have a high negative charge or possess protein coronas, they can be recognized by the immune system. If nanoceria are very small, they can penetrate hepatocytes by passing through cell membranes [88]. However, determining the exact size thresholds is difficult, as it depends on the type of nanoparticle and the presence or absence of inflammatory processes (since permeability increases [88,89]). This further complicates the study of nanoceria effects, as not only do the characteristics of nanoceria influence its distribution, but biological fluids also alter its characteristics [90]. Artificial gastrointestinal and pulmonary fluids caused partial loss of the citrate coating, particle agglomeration, formation of a protein corona, and changes in the zeta potential of the nanoceria. Although this did not affect the cell metabolism of A549 and Caco-2 cells in vitro, it does not mean that the biodistribution of the nanoparticles remains unchanged.

Moreover, although some studies have examined the distribution of nanoceria after oral administration, nanoceria were not detected in the liver, confirming the poor absorption of nanoparticles from the gastrointestinal tract. Trace amounts of cerium were found in the lungs, but these were most likely due to the aspiration of nanoparticles (methodological artifact) [79,91]. The results of these studies highlight the limitations of this delivery route for achieving systemic effects. These findings underline the need for future oral administration studies with stricter gavage controls to reliably assess gastrointestinal absorption of nanoceria.

### 3.2. Assessment of the Toxicological Impact of Nanoceria on Various Organ Systems

**Figure 2:**
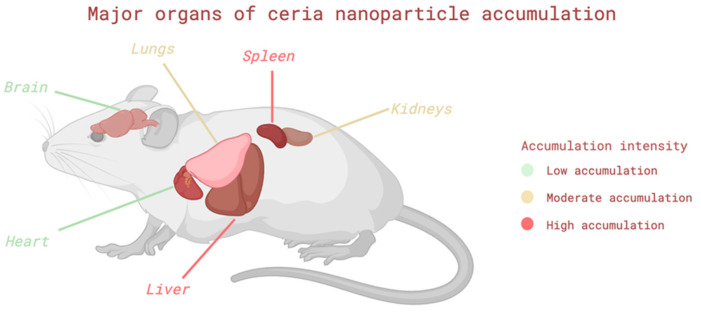
The main organs of nanoceria accumulation in mice in vivo. The color scale reflects the accumulation intensity according to the data of distribution studies (high—liver, spleen; moderate—kidneys, lungs; low—brain, heart). The illustration was created using the BioRender platform.

Nanoceria exhibit pronounced biological activity; however, their impact on the body can range from protective to toxic. The nature of the response is determined by dose, physicochemical properties, and the route of administration, which influences particle accumulation and elimination. Figure 2 shows the primary organs of nanoceria accumulation in mice under in vivo conditions. To assess safety, it is crucial to consider organ-specific reactions and identify damage mechanisms. This section presents experimental data on the TI of nanoceria on various organ systems in healthy mice, enabling the identification of key risk factors and potential limitations for their application (Table 2). Most studies demonstrated low or probably low risk of bias according to the OHAT tool, supported by clear ethical approval, standardized exposure protocols, and parallel control groups. A few studies were rated as probably high mainly because randomization and blinding were not described, and in some cases, the sample size was small or uneven across groups. These limitations reflect incomplete reporting rather than major methodological weaknesses. Overall, the reviewed in vivo and ex vivo studies can be considered methodologically robust, with a generally low risk of bias and good internal validity.

### 3.3. Respiratory System

In the aforementioned study, nanoceria sized 15–30 nm were administered via inhalation to CD-1 mice *(n* = 72) at a dosage of 2 mg/m^3^ for 6 h per day over 7, 14, and 28 days (a total of 24 or 32 exposure days). The investigated pulmonary and extrapulmonary effects were assessed on days 7, 14, and 28 of inhalation and on days 14 and 28 of the recovery period [81]. The pulmonary effects of nanoceria were evaluated using bronchoalveolar lavage (BAL) parameters. The results demonstrated that nanoceria exhibit cytotoxicity (cell viability decreased with prolonged inhalation and recovered slowly after cessation but remained below normal values), induce inflammation (neutrophilia and lymphocytosis were observed against a background of reduced macrophage levels, along with increased levels of TNF-α, IL-1β, and IL-6 during the inhalation period, followed by their gradual recovery), damage lung tissue (a rise in LDH was recorded), and impair alveolar capillary membrane permeability (indicated by an increase in total protein). The authors also recorded an increase in MDA, which is a marker of lipid peroxidation and consequent membrane damage, and a decrease in glutathione, which is a key antioxidant; its depletion indicates a high free radical load. Thus, nanoceria causes oxidative damage to the lungs. In the post-inhalation period, MDA partially decreased, but glutathione did not recover, indicating a prolonged disruption of redox balance in the lungs. Histological examination revealed enlarged mediastinal lymph nodes with accumulation of macrophages laden with cerium particles. This active response of the lymphatic system may suggest possible immunomodulatory effects of nanoceria. Inflammatory and degenerative changes were identified in the lung tissue. As early as day 7 of inhalation, neutrophilic perivascular and peribronchial infiltrates were observed, with macrophages containing nanoceria aggregates. Against the background of inflammation, alveolar deformation and expansion, thickening of interalveolar septa, and destructive changes in the bronchiolar epithelium developed. By day 28, granulomas had formed, consisting of macrophages, epithelioid cells, and giant multinucleated cells, with intracellular nanoparticles. Fibrosis with collagen deposition and fibroblast proliferation developed around the granulomas. Foci of coagulative necrosis were detected in some areas. These morphological changes confirm the data on significant inflammation, damage, and partial irreversibility of processes in lung tissue following prolonged inhalation of nanoceria.

Simultaneously, the accumulation of nanoceria in the lungs is rapid and pronounced (up to 1800 µg/g of tissue), while their elimination is slow: approximately 55% of the accumulated nanoceria remain in the lung tissue 28 days after the cessation of inhalation. Furthermore, the concentration decay curve is not exponential; the decrease in nanoceria slows over time, which may indicate particle redistribution within tissues or the formation of stable intracellular aggregates (e.g., within macrophages or fibrotic tissue). This suggests that the elimination of nanoceria does not follow a classical elimination model, and their behavior in lung tissue is more complex than that of rapidly soluble substances, which is crucial for assessing the long-term toxicity and accumulation risks of nanoceria in the body.

Similar results were obtained in a study investigating the mechanisms of cerium-induced pulmonary fibrosis [92]. A single oropharyngeal instillation of nanoceria at doses of 5 and 50 µg induced dose-dependent development of pulmonary fibrosis in C57BL/6 mice. More pronounced changes, with thickening of alveolar and bronchiolar walls, were observed at the 50 µg dose, starting 24 h after administration. It was shown that nanoceria are actively phagocytosed by alveolar macrophages and induce autophagy. However, blocking autophagy in macrophages (in a group of transgenic mice) protected against the development of alveolar, but not bronchiolar, fibrosis. These findings indicate a key role of macrophages and their autophagic activity in the pathogenesis of cerium-induced fibrosis. These results underscore the potential risks associated with the use of nanoceria via inhalation exposure.

In another study, the behavior of nanoceria in mouse lungs was evaluated after a single intratracheal instillation at a dose of 162 µg [93]. The particles persisted in the lung tissue for up to 180 days, primarily localized within alveolar macrophages. According to microscopy and ICP-MS analysis, the nanoceria underwent local transformation: the average particle diameter decreased from 35 nm to 25 nm, which may indicate partial dissolution within the tissues. Meanwhile, cerium concentrations in the lungs remained statistically significantly elevated throughout the observation period. Although lung histology sections were obtained and stained, their analysis was performed solely for the purpose of visualizing and localizing the nanoparticles; a morphological assessment of potential toxic changes (inflammation, damage, tissue remodeling) was not conducted in this work, which, in this case, limits the ability to draw definitive conclusions about the safety of nanoceria for lung tissue in clinical practice.

A single intratracheal instillation of nanoceria (20 nm) at higher doses (0.1 and 0.5 mg/kg) caused pronounced acute inflammation in the lung tissue of BALB/c mice, characterized by infiltration of neutrophils and macrophages, increased TNF-α levels, and decreased antioxidant activity in BAL fluid and plasma [94]. These findings highlight the potential hazard of cerium-containing nanomaterials upon inhalation and the necessity of assessing their vascular effects when considering clinical applications.

Interestingly, the shape of nanoparticles with different aspect ratios significantly influences their toxicity and cellular interactions [91]. Comparing hexagonal and rod-shaped nanoceria, the authors found that rod-shaped particles induced a more pronounced inflammatory response in the lung tissue upon aspiration exposure in ICR mice (*n* = 6): levels of TNF-α, CXCL-1, and MIP-1α were higher for rod-shaped particles compared to hexagonal ones. However, both forms caused a dose-dependent increase in neutrophil count and a decrease in macrophage count, as well as a rise in pro-inflammatory cytokines in BAL fluid 24 h after aspiration. Histopathological examination revealed inflammatory infiltration and the appearance of giant multinucleated cells in the lung tissue. Thus, the administration of nanoceria into the airways of mice was accompanied by significant acute inflammation of the lung tissue. More pronounced TI was observed with exposure to rod-shaped nanoparticles, indicating the importance of aspect ratio in the pathogenesis of the inflammatory response.

The aim of the next study was to develop an optimal method for visualizing accumulated nanomaterials in the lungs ex vivo, rather than a toxicological assessment of their effects; however, it yielded interesting results [95]. The authors used 3D visualization via micro- and nano-CT, along with histological analysis. Nanoceria were administered at a dose of 50 µg, which is an amount representative for assessing the harm from occupational exposure to metals [96]. Firstly, in situ analysis showed that the nanoceria detected in the lung tissue one week after administration had not undergone biotransformation and remained in the Ce^4+^ form, confirming the high stability of cerium nanoparticles in a biological environment. Secondly, a pronounced heterogeneous distribution of nanoceria was revealed in the lung tissue: along the lumens of the airways and within the alveoli. Moreover, within the alveoli, the distribution remained focal: small aggregates were localized in the alveolar wall and inside macrophages. These observations allow for indirect conclusions about nanoparticle toxicity: phagocytosis by tissue macrophages indicates local irritation or inflammation; heterogeneous distribution may lead to tissue overload and focal damage; and biological stability increases the risk of chronic nanoparticle accumulation in lung tissue. However, it is important to remember that these results are purely morphological and are not supported by biomarkers of inflammation, damage, or fibrosis.

### 3.4. Digestive System

At low concentrations (0.15 and 0.75 mg/kg), nanoceria had a minimal effect on mice [97]. They were orally administered solutions of nanoceria for 10 days, after which the weight of the studied organs (small intestine, large intestine, stomach, liver, spleen, kidneys, lungs, brain, heart) was comparable to control values, and the concentration of nanoceria was below the detection limit of ICP-MS. Consequently, the penetration of nanoceria through the intestinal epithelium is negligible. When assessing the excretion of nanoceria, it is noteworthy that the concentration of cerium in feces after administration of positively charged nanoceria (<25 nm) was significantly higher than after administration of negatively charged nanoceria (30–50 nm), indicating faster gastrointestinal tract (GIT) excretion of smaller, positively charged particles. Seven days after the last administration, the cerium concentration in feces did not differ significantly from that in the control, so it is most likely that mice required less than a week for the residual excretion of nanoceria. These data suggest that, provided the intestinal barrier function is intact, oral intake of nanoceria of the specified size and dosage poses low risks to the organism. However, the limited absorption of such nanoparticles may reduce their effectiveness for oral drug delivery.

In the already mentioned study, the nanoceria deposition and transformation in liver tissue were studied with different routes of their single administration at a dose of 162 μg [92]. The particles were administered either orally or intravenously. After oral administration, nanoceria were not detected in liver tissue either by ICP-MS or histological analysis, indicating no absorption through the GIT. In contrast, after intravenous administration, the nanoparticles quickly reached the liver, where they persisted throughout the entire observation period (180 days), while demonstrating a decrease in average size from 33 nm (day 1) to 28 nm (day 180), indicating their intratissue transformation.

The data from these studies indicate the safety of nanoceria upon oral administration of low doses: the particles are not absorbed into the systemic circulation and do not accumulate in the liver, which minimizes the risks of TI on the GIT. However, such low permeability and rapid excretion without tissue uptake limit the potential use of nanoceria for oral drug delivery.

A number of studies have demonstrated that intranasal administration of nanoceria leads to their translocation [92,98]. Nanoceria of various sizes (35 nm, 300 nm, and 1–5 μm) were administered to female ICR mice at a dose of 40 mg/kg three times (on days 1, 3, and 5), which led to the translocation of nanoceria to the liver and kidneys and caused morphological signs of toxic damage [98]. Hydropic degeneration of hepatocytes and hemorrhages in kidney tissues were observed in all experimental groups. According to ICP-MS data, cerium accumulation in organs was noted only in the groups with 300 nm and 1–5 μm particles, whereas in the 35 nm group, the cerium level did not differ from the control. The results obtained suggest that the TI of nanoceria can develop regardless of particle size, but the degree of accumulation in organs is size-dependent. In another study, the authors specifically investigated the redistribution of nanoceria to the liver following intratracheal administration of lower doses (162 μg/mouse). The nanoparticles persisted in the lungs for up to 180 days, localized primarily in alveolar macrophages, but over time, they translocated to the liver: after 180 days, an average of 2.87 ± 3.37% of the administered dose was detected there [92]. These data highlight the need for a cautious approach to the use of cerium dioxide in aerosol form or for nasal administration.

The properties of nanoceria depend on its valence (+ 3 or + 4) [79,80,81,82]. Although cerium dioxide solutions are administered, they can transform into Ce^3+^ within organs. A study of local changes in nanoceria in the liver was conducted on ICR mice, with nanoceria administered intraperitoneally [99]. Nanoceria were administered at doses of 0.8 and 4 mg/kg for durations of 28 days (once every 2 days) and 7 days (once daily), respectively. In the liver, nanoceria were found mainly in Kupffer cells and on the periphery of the hepatic lobules. It was found that the proportion of cerium in the form of nanoparticles in the liver was 88.8% (under short-term exposure) and 84.8% (under long-term exposure), indicating no significant transformation of the particles and their high stability. The cerium concentration in the liver under long-term exposure was 4.30 ± 0.22 μg/g, while under short-term exposure it was 0.34 ± 0.06 μg/g, despite the higher total dose in the short-term regimen, confirming a tendency for bioaccumulation. Histological analysis revealed changes: inflammatory infiltration in the short-term group and sinusoidal dilation, hydropic degeneration, and the appearance of binuclear hepatocytes in the long-term group. The obtained data indicate the potential hepatotoxicity of nanoceria with long-term use at higher concentrations.

### 3.5. Urinary System

Nanoceria are excreted through the renal system only if their hydrodynamic size is no more than 5–6 nm [85,86,100]. Since smaller nanoceria are more prone to diffusion, the addition of impurities or an increase in the size of the molecules associated with nanoceria could prevent renal excretion and, consequently, a functional load on the urinary system.

Citrate-coated nanoceria appeared in the urine rapidly, within 5 min of administration, while those coated with PAA appeared later (after 2–4 h) but provided the highest total excretion (>83% by day 7). Thus, the renal excretion pathway for nanoceria occurs when they have a small hydrodynamic size (CeO_2_-PAA 3.9 nm, citrate-CeO_2_ 3.1 nm). Despite the active renal excretion of nanoceria, histological analysis on day 7 after administration revealed no toxicological effects on kidney tissues [85].

No renal excretion was recorded for the CeO_2_-DT10 samples, most likely due to their larger size (12.1 nm), as the glomerular membrane allows the passage of molecules with a diameter of no more than 5–6 nm [100]. Additionally, the DT10 coating makes the nanoparticles more hydrophilic, which prevents their recognition and phagocytosis, reduces binding to blood proteins, and results in these nanoceria circulating in the bloodstream for a longer time [85].

At the same time, several studies did not analyze renal excretion but noted slight accumulation of nanoceria in kidney tissue [77,87]. In the experiment by Ernst, nanoceria with a diameter of 3 nm were administered intravenously; therefore, their deposition in kidney tissue was likely due to excretion through the glomerular filter. In the study by Goujon, nanoparticles with diameters of 27.2 and 31.5 nm were administered intravenously, and the subsequent detection of particles in renal tissue likely reflects systemic redistribution and partial glomerular filtration of smaller or less aggregated fractions.

### 3.6. Visual Organs

The effect of nanoceria on the visual system has been studied primarily on eye models. To date, three in vivo studies on murine models investigating the use of nanoceria for the treatment of dry eye syndrome have been described in the literature [101,102,103]. Two of them used nanoceria-based eye drops with concentrations of 1.72 µg/mL and 3000 µg/mL. The third study performed single intravitreal injections with a nanoceria solution at concentrations ranging from 17.2 µg/mL to 1720 µg/mL.

None of the studies revealed clinical, histological, instrumental, or laboratory signs of toxicity. In the experiments where nanoceria were applied as eye drops, a pronounced therapeutic effect was noted. This effect manifested as a lengthening of tear film breakup time, thickening of the corneal epithelium, an increase in the number of goblet cells in the conjunctiva, and a decrease in the expression of cytokeratin 10 (a sign of reduced squamous metaplasia, which is a characteristic pathological feature of dry eye syndrome) [81,83].

Limitations of these studies include their short observation period: the therapeutic effect was evaluated for only 7 days, and it is unknown whether it persists long-term. Toxicity was assessed for up to 30 days, making it impossible to evaluate the long-term consequences of nanoceria administration. Furthermore, the authors did not analyze the systemic distribution of nanoceria or their accumulation in other organs and tissues and did not assess the systemic immune response (only intraocular inflammatory markers were measured).

Two other in vivo studies were conducted on vldlr^−^/^−^ mice, which have congenital pathological retinal neovascularization. These studies do not reflect nanoceria toxicity under physiologically normal conditions and therefore were not included in the main analysis. Nevertheless, in these studies, a single intravitreal injection of nanoceria caused sustained suppression of pathological angiogenesis, reduced OS, decreased apoptosis, and preserved retinal structure and function [66,104]. However, this is rather an assessment of the therapeutic effect of nanoceria, not their toxicity.

Although the review was focused on studies involving somatically healthy animals, the works using the dry eye syndrome model were included in the analysis. This is because this pathology is local in nature, is not accompanied by systemic disorders, and does not affect the general condition of the organism; the inclusion of control groups of healthy mice in the study design allowed for comparison with the physiological norm.

### 3.7. Reproductive System

Toxicity of nanoceria towards the reproductive system has been demonstrated in several experimental studies. The first study evaluated the radioprotective properties of nanoceria in male C57BL/6J mice subjected to local testicular irradiation (2.5–10 Gy) [105]. Intravenous administration of nanoceria (100 µL of a 100 nM solution for 2.5 and 5 Gy irradiation and 100 µM for 10 Gy) reduced the degree of morphological damage, the level of OS (by 13%), and the number of TUNEL-positive cells (by 11%) at doses of 2.5 and 5 Gy. At 10 Gy, the protective effect was weaker and statistically insignificant. No systemic toxicity or lethality was detected.

In another study, male C57BL/6J mice were orally administered cerium dioxide at doses of 10, 20, and 40 mg/kg for 32 days [106]. Dose-dependent, statistically significant accumulation of cerium in the testes was found, along with a reduction in organ weight, sperm motility, and daily sperm production; an increase in DNA damage in germ cells (increase in sperm with single-stranded DNA); and pronounced histopathological changes (atrophy or necrosis of seminiferous tubules, weakening or desquamation of seminiferous epithelial cell adhesion, loss of spermatozoa and apoptosis of interstitial tissue, degeneration of spermatids) at nanoceria doses of 20 and 40 mg/kg. Furthermore, suppression of steroidogenesis enzyme activity, a decrease in testosterone levels (by 14.46% and 33.17% at doses of 20 mg/kg and 40 mg/kg, respectively), and suppression of gene expression regulated by the SF-1 factor were observed. The increase in cerium content in the testes, changes in sperm motility, and daily sperm production were not significant in mice receiving 10 mg/kg of nanoceria (*p* > 0.05). Thus, chronic exposure to nanoceria at doses above 20 mg/kg administered intravenously has a TI on the male reproductive system, including the suppression of sex hormone synthesis and the expression of genes encoding necessary synthesis enzymes.

In a similar study, male BALB/C mice were intraperitoneally administered nanoceria at doses of 100, 200, and 300 µg/kg three times a week for 5 weeks [107]. Dose-dependent decreases in sperm motility and count were revealed. At doses of 200 and 300 µg/kg, levels of FSH, LH, and prolactin decreased, while testosterone levels decreased only at the 100 µg/kg dose and, conversely, increased (though statistically insignificantly) at 300 µg/kg. An increase in morphological abnormalities of the sperm head and tail (up to 44.6% in the 300 µg/kg group) was also observed. Administration of nanoceria led to an increase in MDA and nitric oxide levels and a decrease in the activity of antioxidant enzymes (SOD, glutathione peroxidase, glutathione-S-transferase, and reduced glutathione), indicating significant OS. Furthermore, a significant decrease in hemoglobin (up to −23.1%), hematocrit (up to −18.3%), and red blood cells (up to −28.3%) compared to the control was recorded. Histological examination revealed degeneration and necrosis of spermatogenic epithelial cells, destruction of seminiferous tubule walls, and interstitial changes. This study further confirms the TI of nanoceria on the male reproductive system, mediated through mechanisms of OS, hormonal imbalance, inflammation, and hematological disorders.

In a model of pregnant mice with induced gestational diabetes mellitus, it was shown that intraperitoneal administration of nanoceria (60 mg/kg) reduced OS parameters and decreased the frequency of pathological changes in embryos, reflecting a protective antioxidant effect in a specific disease context rather than toxicity under physiologically normal conditions [108]. In the control group of healthy pregnant mice, administration of nanoceria revealed no deviations in body weight, embryo weight, glucose levels, or OS markers (MDA, reduced glutathione, catalase, protein carbonyls) compared to intact control mice. Histological examination revealed no pathological changes in the embryos. However, it is impossible to conclude that there were no toxic effects of nanoceria detected under physiological pregnancy conditions in mice, since the authors did not provide data on the size of nanoparticles. Theoretically, the lack of effect in healthy pregnant mice could be due to the fact that the nanoparticles did not penetrate the placental barrier [109].

### 3.8. Nervous System

Analysis of available in vivo studies in mice indicates that nanoceria are capable of penetrating the BBB. In an experiment on SJL/J mice (*n* = 10), it was found that nanoceria sized 2.9 nm, stabilized with a combination of citrate and EDTA, after a single intravenous administration at a dose of 20 mg/kg, were not only able to cross the BBB but also accumulated in brain tissues [82]. The cerium content in the brain remained measurable for at least five months after administration, indicating long-term persistence of the particles in the central nervous system (CNS). Morphological and functional assessment of the nervous system state was not performed in this series of experiments. However, another study, where healthy mice were used to compare different forms of nanoceria, showed that stabilization of nanoparticles with citrate and EDTA prevents their aggregation, promotes longer circulation in the bloodstream, and facilitates penetration into the brain [83]. In contrast, non-stabilized particles or particles stabilized only with citrate were detected in CNS tissues only in trace amounts.

From a biomedical perspective, these results indicate the potential for delivering nanoceria to brain tissues, which could be valuable for developing antioxidant and neuroprotective therapies. However, the lack of data on neurotoxicity under physiological conditions requires further safety investigations.

### 3.9. Hematopoietic System

It is known that rare earth metals, including cerium, possess anticoagulant properties by acting as calcium inhibitors and reducing the activity of thrombin and activated factor X [110].

In an experiment on BALB/c mice, intratracheal administration of nanoceria at doses of 0.1 and 0.5 mg/kg caused a dose-dependent reduction in the time to thrombotic occlusion (the interval from vascular wall injury to complete vessel occlusion by a thrombus) in arterioles and venules of the pia mater after 24 h. This was accompanied by an increase in plasma concentrations of fibrinogen and plasminogen activator inhibitor-1 [93]. However, the direct addition of nanoceria (1–25 µg/mL) to whole blood in vitro did not induce platelet aggregation or alter prothrombin time (PT) and activated partial thromboplastin time (aPTT) values. This suggests that the prothrombotic effect is mediated, likely through systemic inflammation and OS.

The literature describes a single clinical case of acute oral poisoning with nanoceria (99.99%, 20–40 nm) in a human [111]. A 30-year-old woman accidentally drank about 1000 mL of water (two portions of 500 mL each) containing a sediment of cerium dioxide used as a polishing agent in her workplace. The water had no taste or smell, so the woman did not notice the impurity. Ten hours after ingestion, the patient developed multiple petechiae on her face, neck, and limbs. Laboratory tests revealed decreased activity of plasma factors VII, VIII, IX, XI, and XII, prolonged aPTT, and reduced fibrinogen concentration, while PT and platelet count were normal. Cerium was detected in the blood and urine (47 µg/L and 542 µg/L, respectively). The administered treatment (forced diuresis, laxatives, sodium dimercaptopropanesulfonate, dexamethasone) led to the normalization of coagulation parameters by day 7 and the disappearance of clinical manifestations. Although this case occurred in a human and not in a mouse experiment, it is of significant interest, as it confirms the possibility of coagulation disorders following the acute intake of nanoceria.

In a series of in vivo experiments studying the radioprotective properties of nanoceria, their effect on the hematopoietic system was evaluated in control groups without irradiation [112,113,114]. One study used citrate-stabilized nanoceria (3–4 nm), which were administered to mice either intravenously or intraperitoneally in a single dose [113]. Analysis of the bone marrow in non-irradiated animals revealed no morphological changes compared to the control. In another study, nanoceria (2 nm) was administered intravenously in single doses of 4–12 mg/kg [112]. After 7 days, non-irradiated animals showed normal counts of bone marrow nucleated cells, histological structure, and distribution of cells across all hematopoietic lineages. In both cases, the administration of nanoceria caused no deviations from the intact control values, indicating the absence of TI on the hematopoietic system of healthy mice. In the irradiated groups, however, both types of nanoparticles demonstrated significant protective properties, reducing damage to hematopoietic tissue and accelerating bone marrow recovery. These data indicate that the studied forms of nanoceria at the administered doses are safe for the hematopoietic system of healthy animals and, at the same time, possess pronounced radioprotective potential. This makes them promising for the development of agents to prevent and correct radiation-induced suppression of hematopoiesis.

### 3.10. Immune System

In the spleen, as a primary immune organ, the distribution characteristics and potential impact of nanoceria following intraperitoneal administration were evaluated [99]. According to the obtained data, under short-term exposure (4 mg/kg once daily for 7 days), the nanoparticles were localized primarily in the marginal zone. In contrast, under long-term administration (0.8 mg/kg once every 2 days for 28 days), they were also found in the white pulp. ICP-MS analysis showed a significant increase in cerium content after long-term exposure (9.07 ± 0.08 µg/g vs. 1.40 ± 0.17 µg/g), with 97.9% of the cerium remaining in nanoform. In certain areas of the white pulp, an increase in Zn concentration was noted, which may indicate the activation of zinc-containing proteins. No histological changes in the spleen structure were recorded; however, TEM revealed nanoparticle deposits in the marginal zone and the presence of eosinophils in the white pulp, potentially reflecting a local immune response. These results demonstrate the long-term persistence of nanoceria in lymphoid tissue and a possible immunomodulatory effect upon accumulation in functionally active zones of the spleen.

The inclusion of the following study in the review is justified because, despite using a model of acute induced paw inflammation (rather than physiologically healthy animals), it provides important data on the effect of citrate-stabilized nanoceria on the immune system and their toxicological assessment [115]. In an in vivo experiment on C57BL/6J mice, intravenous administration of nanoceria at a dose of 100 mg Ce/kg, 24 h after inflammation induction, reduced edema, pain sensitivity, infiltration by CD68-positive macrophages, and levels of TNFα and IL-1β, but increased the expression of IL-10 in the affected tissue. The authors also investigated pharmacokinetics: nanoceria had a short half-life (29 min) and effective renal excretion (less than 16% of the administered nanoparticle dose remained in the body after 24 h). Histological examination of the heart, lungs, liver, spleen, and kidneys, as well as biochemical indicators of liver and kidney function, revealed no signs of acute toxicity. Thus, citrate-stabilized nanoceria effectively suppress acute inflammation with good tolerability and rapid excretion, making them promising for the development of safe anti-inflammatory drugs.

### 3.11. Cardiovascular System

Only one of the identified studies assessed the effect of nanoceria (7 nm, 0.15 mM in a 100 µL solution) administered intravenously twice a week for 2 weeks in a model of cardiomyopathy in transgenic mice with cardiac-specific expression of MCP-1 [116]. The animals were divided into three groups: transgenic mice receiving nanoceria, transgenic mice receiving saline solution, and healthy wild-type (WT) mice receiving nanoceria or not receiving them. Untreated transgenic mice exhibited progressive left ventricular dilation and dysfunction, pronounced inflammatory infiltration, and increased expression of pro-inflammatory cytokines, OS markers, and ER-stress genes. Administration of nanoceria in this group significantly reduced the severity of these pathological changes. In healthy WT mice receiving nanoceria, no deviations were observed compared to the control group receiving saline solution, indicating the absence of TI of nanoceria on the heart in this study. Limitations include the fact that the authors did not determine the hydrodynamic particle size and stability in the solution, which could affect the biodistribution and biological activity of nanoceria. Furthermore, the effect assessment was conducted primarily remotely (at 6 months), and periodic echocardiography was not accompanied by an analysis of molecular changes. 

### 3.12. Musculoskeletal System

Only one in vivo study related to the effect of nanoceria on the musculoskeletal system of mice was found in the literature [117]. This study investigated whether nanoceria (0.5 mg/kg, intraperitoneally) could protect the Swiss mice skeletal muscles with diabetes mellitus from ischemia–reperfusion injury. This does not meet the criteria of the current review, as the study was not conducted on healthy animals. Nevertheless, the authors demonstrated that in the group of animals receiving nanoceria, indicators of OS, the severity of muscle fiber damage, and inflammatory infiltration were lower. No other studies on mice without somatic pathologies investigating the TI of nanoceria on the musculoskeletal system were found.

### 3.13. Skin

No studies meeting the inclusion criteria were found for this section. One of the identified studies was conducted in vitro using removed human skin, and another focused on the dermal permeability of cerium salts, not cerium oxide nanoparticles [95,118]. Thus, there is no data on the TI of nanoceria on the skin within the specified framework of this review.

### 3.14. Endocrine System

There is a lack of current studies in the literature assessing the TI of nanoceria on the endocrine system in healthy mice. The available data pertain either to reproductive function (including pregnancy models) or to animals with experimentally induced diseases, which prevents direct extrapolation of the results to the endocrine system under physiologically normal conditions [108,119].

## 4. Discussion

Analysis of the presented studies indicates that the TI of nanoceria is complex and determined by dose, size, route of administration, frequency of application, and the physicochemical properties of the particles. The most vulnerable targets for nanoceria accumulation are the liver, spleen, lungs, and kidneys; therefore, these organs require special monitoring when assessing the safety of nanoceria. Evidence from multiple studies confirms that nanoceria, when administered at doses ≤ 5 mg/kg and particle sizes of 5–8 nm, does not elicit morphological or functional impairments, demonstrating a potential safety profile.

For oral delivery, a daily dose of ≤1 mg/kg did not produce detectable toxicity, whereas single doses up to 10 mg/kg were non-lethal but caused biochemical and histological signs of inflammation in the liver and kidneys. Although these doses may lead to cerium accumulation in target organs, the observed morphological changes were mild and not associated with overt tissue damage.

Single parenteral injections of ≤5–10 mg/kg do not lead to acute tissue damage: the nanoparticles persist in tissues for a long time without signs of acute inflammation or necrosis. When nanoparticles directly enter the bloodstream or abdominal cavity, a significant portion accumulates in filtering organs (the liver, spleen, and partly the kidneys and lungs), which creates a potential hazard. However, with low doses and correct particle characteristics, no acute damage is observed. The intravenous route is more suitable for controlled nanoparticle application but requires dose limitation to prevent accumulation in organs and subsequent damage.

It should be emphasized that the findings are not uniform: some studies reported protective effects of nanoceria (antioxidant, neuroprotective, radioprotective), whereas others demonstrated toxic outcomes (pro-oxidant and cytotoxic). This dual behavior depends on dose, particle size, administration route, and model characteristics and should be considered when interpreting the results and designing future studies.

Transitioning to intra-organ routes of administration results in more pronounced TI: acute and chronic inflammation, fibrosis, OS, and immune and hormonal disorders can develop. For instance, intraperitoneal administration of even very low doses of nanoceria (100 µg/kg) causes systemic disturbances in the organism. However, this route of administration is generally relatively safe, as even higher doses (up to 10 mg/kg) do not cause signs of acute toxicity, and doses in grams per kg do not lead to instant death or severe acute reactions. Safe levels of inhalational exposure to nanoceria have not been precisely established, as experimental data indicate toxicity even at relatively low aerosol concentrations. For example, chronic inhalational exposure to 2 mg/m^3^ (6 h/day, up to 28 days) led to significant lung damage, development of inflammation, necrosis, and fibrosis, as well as renal changes (tubular necrosis), despite the absence of pronounced liver dysfunction. Nanoparticles of 15–30 nm accumulated in lung tissue in large quantities (1800 µg/g) and were slowly eliminated: 55% of the accumulated cerium remained in the lungs 4 weeks after exposure ceased. Thus, constant inhalation of even a few mg/m^3^ for weeks causes serious damage, indicating the absence of a “safe” dose within the studied range and the necessity to minimize inhalation of nanoceria. Permissible exposure levels are likely an order of magnitude lower than 2 mg/m^3^, but this requires further investigation. Inhalation of nanoparticles causes direct TI on the lungs; therefore, respiratory routes of administration are associated with the highest risk of toxicity. Even small amounts of nanoceria can provoke local lung lesions and mediate systemic effects. These routes require the most thorough safety assessment, and their own maximum permissible doses, likely much lower than for parenteral or oral administration, should probably be established. However, to date, data are insufficient, and any highly dispersed forms of cerium dioxide should be considered potentially dangerous upon entry into the respiratory tract.

Application of nanoceria on the eye surface (as drops) or their injection into the vitreous body demonstrated the absence of local toxic reactions. This indicates that with local use (without entry into the systemic bloodstream), the nanoparticles can be quite safe, especially considering their antioxidant effect, which is beneficial for tissues. In particular, nanoceria doses up to 0.3% (3000 µg/mL) applied topically or intraocularly can be considered safe for the eyes. Similarly, dermal application of nanoceria (e.g., on a wound or skin) in the absence of systemic absorption is expected to be relatively safe, although the review did not include studies on dermal application due to the lack of suitable in vivo studies for skin. Thus, local routes of administration potentially have the lowest risk of toxicity, but their safety and efficacy are limited to the application area.

Regarding characteristics, the most toxic to living systems are nanoceria with a positive charge, an unstable, agglomerating surface (without coating), high stability (low solubility, remaining in the Ce^4+^ form), and an elongated shape. The combination of these traits (e.g., cationic particles of medium size without coating, aggregating to large sizes) appears to be the worst-case scenario from the toxicology perspective of nanoceria. Optimizing their characteristics can reduce the toxicity of nanoceria. The following parameters are considered optimal for safety and preservation of therapeutic effect: size of 2–6 nm, negative charge, biocompatible coating, balanced Ce^3+^/Ce^4+^ ratio, and spherical shape. The combination of these factors, for example, particles sized 2–5 nm stabilized with a negatively charged hydrophilic biopolymer (PAA, PEG, citrate, etc.), demonstrates the best safety in experiments.

Nanoceria are relatively safe with rare or single administrations, when the organism has time to eliminate most of the particles without significant accumulation in organs. Repeated administration, especially at short intervals, significantly increases toxicity, even if the single doses are small (0.2 mg/kg and above). Safe regimens can be considered as single-use or short courses with long rest periods (about 7 days), allowing for the elimination of the cerium accumulated by that time. Thus, the dosing regimen should be gentle: the minimum effective dose and the lowest frequency of administration ensure better tolerability.

The high biopersistence of nanoceria and the identified subclinical changes upon long-term exposure indicate the necessity for further long-term studies and standardization of nanoparticle characteristics for reliable safety prediction before clinical use.

## 5. Conclusions

The totality of the obtained data in murine models emphasizes that under the specified conditions (prudent selection of dose, size, coating, and routes of administration; limitation of frequency; or long pauses between doses), nanoceria are safe and can be applied without significant side effects. Based on the analyzed studies, CeO_2_ NPs sized ~2–10 nm and dosed up to 5 mg/kg demonstrated a favorable safety profile in healthy murine models, provided that coating/charge is optimized and dosing is infrequent or spaced by long intervals. The local application of nanoceria has the lowest risk of toxicity.

### 5.1. Limitations

Several limitations should be considered when interpreting the results of this systematic review. First, we limited our review to in vivo studies in healthy mice. In vitro and non-mouse studies were excluded. Second, the review included the latest studies published mainly after 2019. We deliberately restricted the search to 2019–2025 in order to focus on the most recent data and to provide the most up-to-date and comparable picture of the toxicological effects of CeO_2_ in healthy murine models. During this period, updated standards for preclinical reporting (ARRIVE 2.0 [120]) and advanced tools for nanomaterial safety assessment [121] were widely adopted. Earlier studies had some basic methodological disadvantages: small sample size without adequate statistical analysis, the absence of control groups or their insufficient characterization, the use of inadequate methods of quantitative analysis, as well as an incomplete description of the characteristics of nanoparticles (size, charge, coating, and synthesis method). Such limitations did not allow for reliable conclusions about the biodistribution and toxicological impact of cerium oxide nanoparticles, so in order to form a more reliable database, the analysis included mainly modern publications from recent years that meet higher quality standards. Although some of the latest studies still have limited sample sizes and heterogeneous designs as the most significant methodological limitations, the quality of the 2019-2025 studies included in the analysis is much higher. In addition, the overall methodological quality and potential sources of bias were qualitatively assessed in accordance with the OHAT’s risk of bias tool for animal studies [122]. Several of the included studies lacked detailed descriptions of randomization, blinding, and allocation concealment procedures, and some did not specify sample size justification. These factors indicate potential sources of bias that may influence the robustness of the reported outcomes. Overall, most included studies demonstrated low or probably low risk of bias, supported by ethical approval, standardized exposure protocols, and clearly defined control groups. Nevertheless, the overall internal validity of the included evidence can be considered high, and the results are suitable for qualitative synthesis within this systematic review. Finally, we could not include in the review publications that were incomplete or inaccessible in English, which may have introduced a language (or geographical) bias.

### 5.2. Prospects

The obtained data indicate that further research on nanoceria requires a systematic approach and more stringent methodological standards. An important area of research is conducting studies with large samples, properly selected control groups, and long-term experiments with extended follow-up periods. This will allow for the assessment of the accumulation and possible chronic effects of nanoceria, improving the reliability and reproducibility of the data obtained. Special attention should be paid to standardizing the characteristics of nanoparticles, such as size, shape, coating, charge, and synthesis method, as these parameters largely determine their biological properties and activities. Comparative studies of different routes of administration (intranasal, oral, intravenous) and their contribution to systemic accumulation are needed. In addition, experiments should be expanded to longer-lived models and different animal species to assess interspecies differences. From a translational perspective, it is advisable to further explore the therapeutic potential of CeO_2_ NPs in conditions associated with oxidative stress and inflammation, including neurodegenerative diseases and cardiovascular pathologies. However, the transition to clinical trials requires a prior accumulation of robust safety information and the development of unified toxicity assessment standards.

At the same time, it can be stated that the use of nanoceria does not have obvious toxicity, while useful biological effects are observed. This means that it is promising to develop a fundamentally new type of medicine based on nanoceria.

## Figures and Tables

**Table 1:** Systemic toxicity of cerium dioxide nanoparticles: summary of in vivo studies included in the current review.

Title of the Article	Authors	Year	DOI	Type of Experiment	Mouse Strain	Number of Mice (P+C)	Control Group	Nanoceria Size	Nanoceria Dose	Route of Administration	Frequency of Administration	Assessment at [Number] Days Post-Administration	Route of Elimination	Organs with the Highest Concentration	Key Findings	Substance	OHAT Risk of Bias Tool
Anti-inflammatory properties of cerium oxide nanoparticles	Suzanne Marie Hirst et al.	2009	10.1002/smll.200901048	in vivo	C57BL/6	24 (16 + 8)	Yes, saline solution	3–5 nm	0.1 mg/kg 0.5 mg/kg	Intravenously	Once; 2 times in 7 days	7, 30 days	Not studied	Liver, Kidneys, blood vessels	When administered intravenously, NPs are capable of persisting for a long time in the body’s tissues without inducing acute damage.	CeO_2_ NPs	Probably low
Biodistribution and in vivo antioxidant effects of cerium oxide nanoparticles in mice	Suzanne Marie Hirst et al.	2011	10.1002/tox.20704	in vivo	CD-1	48 (36 + 12)	Yes, saline solution	3–5 nm	0.5 mg/kg	Orally Intravenously Intraperitoneally	Once a week for 2 or 5 weeks	3, 24 h, 2, 5 weeks	Hepatobiliary	Lungs	NPs are distributed in the spleen, liver and lungs, excreted with feces, and demonstrate antioxidant properties. The greatest deposition of NPs was due to intravenous and intraperitoneal administration. During the study, cerium was practically not excreted from the organs under study.	CeO_2_ NPs (carboxyfluorescein coating)	Probably low
												24 h, 2 weeks		Spleen, liver, Lungs, kidneys, heart, Brain			
												24 h, 2 weeks					
Intrinsically Radiolabeled Multifunctional Cerium Oxide Nanoparticles for in vivo Studies	Likun Yang et al.	2013	10.1039/c2tb00404f	in vivo and ex vivo	Nude	36	No	2 nm	140 pmol/mouse	Intravenously	Single dose	5 min, 1, 4, 24, 48, 120 h	Renal	Kidneys, liver, spleen, lungs	The NP rate and route of excretion depend on their size (2 nm via the kidneys within 10 min, 6 nm via the liver, T1/2 = 27 min).	PAA rCONP	Probably low
								6 nm					Hepatobiliary			DT10 rCONP	
In Vivo Inflammatory Effects of Ceria Nanoparticles on CD-1 Mouse	Anna Poma et al.	2014	10.1155/2014/361419	in vivo	CD-1	3	No	5 nm	2000/3500/5000 mg/kg	Orally	Single dose	10 days	Not studied	Lungs, Liver	NPs were not lethally toxic in either acute or subacute tests. However, they caused inflammatory changes in the liver, kidneys, and lungs. The most pronounced inflammation was observed at low and medium doses, which may be due to better bioavailability of the nanoparticles. High doses could cause aggregation of the NPs, which reduces their toxic effect.	CeO_2_ NPs	Probably low
						24 (18 + 6)	Yes, saline solution		50/500/5000 mg/kg	Intraperitoneally		14 days		Liver, kidneys, lungs			
Toxicity and bioaccumulation of inhaled cerium oxide nanoparticles in CD1 mice	Srinivas Aalapati et al.	2014	10.3109/17435390.2013.829877	in vivo	CD-1	72 (66 + 6)	Yes, without impact	15–30 nm	2 mg/m^3^	Inhalation	Inhalation 6 h a day	7, 14, 28 days	Mucociliary	Lungs, kidneys, liver, Heart, brain, lymph nodes	Inhalation of NPs causes inflammation, necrosis and fibrosis in the lungs. The kidneys are damaged (tubular necrosis), while the liver retains its functions. Elimination is slow: mainly through mucociliary clearance and then through ingestion and the gastrointestinal tract; renal excretion is limited. NPs have high biopersistence.	CeO_2_ NPs	Probably low
Custom cerium oxide nanoparticles protect against a free radical mediated autoimmune degenerative disease in the brain	Karin L. Heckman et al.	2013	10.1021/nn403743b	in vivo	C57BL/6	10 (exact number for C not specified)	Yes, saline solution	2.9 nm	20 mg/kg	Intravenously	Not exactly described (probably on (3), 7, 14, 21, 28, 35 days)	24 h; 1, 2, 3, 4, 5 months	Hepatobiliary	Brain, Liver, spleen, kidneys	Using mass spectrometry to analyze the accumulation and effectiveness of NPs in the body, antioxidant activity has been demonstrated.	CA/EDTA CeO_2_ NPs, CeO_2_ NPs	Probably low
Nanoceria distribution and effects are mouse-strain dependent	Robert A. Yokel et al.	2020	10.1080/17435390.2020.1770887	ex vivo	C57BL/6, BALB/c	80 (60 + 20)	Yes, saline solution	4.2 ± 1.2 nm	10 mg/kg	Intraperitoneally	Single dose	0.5, 1, 3, 6, 24 h	Hepatobiliary	Liver, spleen, kidneys, lungs, heart, Brain	C57BL/6 mice showed greater accumulation of NPs in the body. BALB/c mice had more pronounced inflammation. Intracellular nanoparticles were bioprocessed to form crystalline cerium phosphate nanostructures.	CeO_2_ NPs (citrate coating)	Definitely low
Biodistribution and PET Imaging of 89-Zirconium Labelled Cerium Oxide Nanoparticles	Philip Reed McDonagh et al.	2018	10.1016/j.nano.2018.04.002	in vivo and ex vivo	C57BL/6	23–41 (exact number not specified)	^89^ZrCl_4_ in saline	Without coating: 5 nm citrate: 3.1 ± 0.5 nm DT10-NH_2_: 6.3 ± 1.8 nm DT10-PEG: 9.3 ± 2.7 nm DT10-SB: 14.3 ± 4.6 nm PAA: 3.9 ± 0.9 nm	0.37 MBq/mouse 3.7 MBq/mouse 11.1 MBq/mouse	Intravenously, Orally, Intraperitoneally	Single dose	2, 24 h, 7 days	Renal, hepatobiliary	Liver, spleen, kidneys, lungs	The coating of NPs significantly affects their biodistribution and elimination. PAA-NPs show better renal elimination (>75% in 4 h), while uncoated NPs are retained in the lungs and are slowly eliminated (<3% in 7 days). DT10-PEG prolongs circulation in the bloodstream, and DT10-NH_2_ is rapidly taken up by the liver. The importance of coating is critical for biomedical applications.	CeO_2_ NPs PAA-CeO_2_ NPs Citrate-CeO_2_ NPs DT10-PEG-CeO_2_ NPs DT10-NH_2_-CeO_2_ NPs DT10-SB-CeO_2_ NPs	Definitely low
In vivo-induced size transformation of cerium oxide nanoparticles	JustynanModrzynska et al.	2018	10.1371/journal.pone.0202477	in vivo	C57BL/6	108 (81 + 27)	Yes, saline solution	13.0 ± 12.1 nm	162 μg per mouse	Intratracheally, intravenously, Orally	Single dose	1, 28, 180 days	Not studied	Lungs, Liver	NPs after entering the lungs are slowly transported to the liver, where they accumulate in Kupffer cells. Their size decreases over time (intra-organism transformation), but this does not affect excretion. After 180 days, a significant number of NPs remain in the lungs and liver, which confirms the low rate of excretion and long-term accumulation in the body. They are not absorbed from the gastrointestinal tract.	CeO_2_ NPs	Definitely low
In vivo toxicological evaluation of polymer brush engineered nanoceria: impact of brush charge	JuliaCatalán et al.	2019	10.1080/17435390.2018.1543469	in vivo	C57BL/6	72 (54 + 18)	Yes, saline solution, Tween-80, methyl methanesulfonate	CeO_2_ (80–150 nm) PAA (20 nm) PMETAC (10 nm)	1.8 mg Ce/kg 5.3 mg Ce/kg 16.0 mg Ce/kg	Intraperitoneally	4 injections with an interval of 4–5 days	15 days	Not studied	Liver, spleen, bone marrow	Coating of nanoceria with positively charged polymer brushes (CeO_2_@PMETAC) induced organ inflammation, cerium accumulation and hematological changes, while CeO_2_ and CeO_2_@PAA did not cause significant toxicity or genotoxicity.	CeO_2_ NPs	Probably low
Antioxidant Activity and Toxicity Study of Cerium Oxide Nanoparticles	Geoffroy Goujon et al.	2021	10.1002/adhm.202100059	in vivo	Swiss	15 (6 + 9)	Yes, saline solution	Core size: 9 nm; hydrodynamic diameter (P1): 27.2 nm; (P3): 31.5 nm	5 mg/kg	Intravenously	Single dose	5, 15, 30 min; 1, 3, 6 h; 1, 2, 3, 7, 14, 21, 28 days	Hepatobiliary	Spleen, liver, kidneys, lungs, Brain	P3-coated NPs showed antioxidant activity in endothelial cells (reduction in DNA oxidation and superoxide anion production). The main accumulation organs are the liver and spleen (max concentration 30 min after administration). After 14 days, the NPs level decreased almost to background levels.	CeO_2_ NPs (P1—coating with phosphonic acid and methyl PEG chains. P2—includes phosphonic acid, methyl PEG chains and amino-Peg 1000. P3—similar to P2, but with amino-Peg 2000.)	Probably low
Exploring the Long-Term Tissue Accumulation and Excretion of 3 nm Cerium Oxide Nanoparticles	Lena M. Ernst et al.	2023	10.3390/antiox12030765	in vivo	BALB/C	30	No	3 nm	5.7 mg/kg	Intravenously	Single dose	1, 9, 30, 100 days	Hepatobiliary	Liver, Spleen	The distribution of NPs depends on their characteristics: when captured by resistant macrophages, they can remain there until they are broken down into ions and then excreted via the urinary tract, while hepatocytes subject NPs to biliary excretion.	CeO_2_ NPs (coated with mouse serum albumin (MSA))	Definitely low

**Table 2:** Summary of in vivo toxicity studies of cerium dioxide nanoparticles on organs and systems.

Organ System	Title of the Article	Author	Year	DOI (PMID if No DOI)	Type of Experiment	Mouse Strain	Number of Mice (P+C)	Control Group	Nanoceria Size	Nanoceria Dose	Route of Administration	Frequency of Administration	Assessment at [Number] Days Post-Administration	Route of Elimination	Substance	OHAT Risk of Bias Tool
Respiratory system	Toxicity and bioaccumulation of inhaled cerium oxide nanoparticles in CD1 mice	Srinivas Aalapati et al.	2014	10.3109/17435390.2013.829877	in vivo	CD-1	72 (66 + 6)	Yes, without impact	15–30 nm	2 mg/m^3^	Inhalation	6 h/day during exposure (7, 14, 28 days)	8 days, 15 days, 29 days	NPs inhalation causes inflammation, necrosis and fibrosis in the lungs, and an active reaction in the lymph nodes.	CeO_2_ NPs	Probably low
	Multi-scale X-ray computed tomography to detect and localize metal-based nanomaterials in lung tissues of in vivo exposed mice	Chaurand P, Liu W, Borschneck D et al.	2018	10.1038/s41598-018-21862-4	ex vivo	C57/Bl6	10 (5 + 5)	5 mice were given water	TEM size: 31 ± 18 nm; hydrodynamic diameter: ≈90 nm	50 μg in 25 μL	Intratracheal instillation	Single dose	7 days	Uneven distribution of CeO_2_-NMs in lung tissue. Accumulations were found in the respiratory tract, parenchyma, and macrophages (size agr. 300–2373 nm). Preservation of CeO_2_ and absence of biotransformation were confirmed.	CeO_2_-NMs (cerium dioxide)	Probably high
	Macrophage autophagy protects mice from cerium oxide nanoparticle-induced lung fibrosis	Annangi B, Lu Z, Bruniaux J, Ridoux A, da Silva VM, Vantelon D, Boczkowski J, Lanone S.	2021	10.1186/s12989-021–00398-y	in vivo	C57Bl/6	5–8 mice per group (4 exposed groups, 2 control groups)	Yes, saline solution	TEM size: 22.4 ± 0.2 nm; hydrodynamic diameter: 1480 nm	5 μg 50 μg	Oropharyngeal instillation	Single dose	24 h, 7 days, 28 days	50 μg CeO_2_ NP induces progressive pulmonary fibrosis. Blockade of macrophage autophagy protects against alveolar but not bronchiolar fibrosis.	CeO_2_ NPs	Probably high
	The acute pulmonary and thrombotic effects of cerium oxide nanoparticles after intratracheal instillation in mice	Nemmar A, Al-Salam S, Beegam S, Yuvaraju P, Ali BH.	2017	10.2147/IJN.S127180	in vivo	BALB/C	6–8 mice in the group (n ≈ 18–24)	Yes, saline solution	20 nm	0.1 and 0.5 mg/kg	Intratracheal instillation (0.1 mL)	Single dose	24 h	A single intratracheal instillation caused acute pneumonia with neutrophil and macrophage infiltration, increased TNF-α, and decreased antioxidant enzyme activity.	CeO_2_ NPs	Probably high
	In vivo-induced size transformation of cerium oxide nanoparticles in both lung and liver does not affect long-term hepatic accumulation following pulmonary exposure	Modrzynska J, Berthing T, Ravn-Haren G, Kling K, Mortensen A, Rasmussen RR, Larsen EH, Saber AT, Vogel U, Loeschner K.	2018	10.1371/journal.pone.0202477	in vivo	C57BL/6	162 (81 + 81)	Yes, without nanoceria administration	TEM core size: 13.0 ± 12.1 nm; hydrodynamic diameter: 79 nm	162 μg	Intratracheal instillation Orally Intravenously	Single dose	1 day, 28 days, 180 days	NPs were detected in the liver of mice 180 days after intratracheal and intravenous administration, where a decrease in their size over time was also observed. After oral administration, NPs were not detected in the liver tissue.	CeO_2_ NPs	Definitely low
Digestive system	Translocation of intranasal (i.n.) instillation of different-sized cerium dioxide (CeO_2_) particles: potential adverse effects in mice	Liu Y, Ji J, Ji L, Li Y, Zhang B, Yang T, Yang J, Lv L, Wu G.	2019	10.1080/15287394.2019.1686867	in vivo	ICR	40 (30 + 10)	Yes, saline solution	35 ± 3 nm 287 ± 27 nm 1–5 μm	40 mg/kg	Intranasal instillation (in one nostril)	3 times (day 1, 3, 5)	7 days	In all groups, pronounced histological changes were revealed: hydropic degeneration in the liver and hemorrhages in the cortex and medulla of the kidneys. According to ICP-MS, cerium accumulation in the liver and kidneys occurred with the introduction of 300 nm and 1–5 μm particles, but not 35 nm. In the 35 nm group, a statistically significant decrease in total bilirubin, total protein and alkaline phosphatase, and an increase in the triglyceride level were observed. In the 1–5 μm group, an increase in AST activity was additionally noted.	CeO_2_ NPs	Probably low
	Fate and distribution of orally-ingested CeO_2_-nanoparticles based on a mouse model: Implication for human health	Ma X, Wang X, Xu L, Shi H, Yang H, Landrock KK, Sharma VK, Chapkin RS.	2023	10.1016/j.seh.2023.100017	in vivo	C57BL/6	48 (36 + 12)	Yes, without nanoceria administration	<25 nm (CeO_2_) 30-50 nm (CeO_2_ coated with polyvinylpyrrolidone), hydrodynamic size 110.8 ± 0.8 nm and 326.4 ± 17.1 nm, respectively	4 µg CeO_2_ 30–50 nm (0.15 mg/kg) 20 µg CeO_2_ 30–50 nm (0.75 mg/kg) 20 µg CeO_2_ <25 nm (0.75 mg/kg)	Orally	Every day for 10 days	½ of mice at 10 days, another ½ of mice in 7 days	Oral administration of NPs at doses of 0.15–0.75 mg/kg did not cause toxicity in mice; nanoparticles did not accumulate in organs and were completely excreted with feces in less than 7 days.	CeO_2_ NPs CeO_2_ NPs coated with polyvinylpyrrolidone	Probably low
Immune system	Integration of sub-organ quantitative imaging LA-ICP-MS and fractionation reveals differences in translocation and transformation of CeO_2_ and Ce^3+^ in mice.	Chen B, Lum JT, Huang Y, Hu B, Leung KS.	2019	10.1016/j.aca.2019.07.044	in vivo	ICR	8 (5 + 3)	Yes, without nanoceria administration	30–50 nm, hydrodynamic size 152.7 ± 1.6 nm	0.8 mg/kg	Intraperitoneally	Once every 2 days (14 injections)	28 days	After intraperitoneal administration of NPs to mice, accumulation of particles was observed in the marginal zone and white pulp of the spleen, as well as in Kupffer cells of the liver; 85–98% of cerium was retained in nanoform, and long-term exposure caused inflammatory changes in the liver and an immune response in the spleen.	CeO_2_ NPs	Probably moderate
	Ultrasmall Antioxidant Cerium Oxide Nanoparticles for Regulation of Acute Inflammation	Kim J., Hong G., Mazaleuskaya L., Hsu J.C., Rosario-Berrios D.N., Grosser T., Cho-Park P.F., Cormode D.P.	2021	10.1021/acsami.1c16126	in vivo	C57BL/6J	32 (16 + 16)	Yes, saline solution	2.8 ± 0.4 nm, hydrodynamic size 3.4 ± 1.1 nm	100 mg Ce/kg	Intravenously	Single dose	3, 6, 18, 24 h	In mice with induced paw inflammation, intravenous administration of NPs rapidly reduced swelling and pain, decreased CD68, TNFα and IL-1β, increased IL-10 and did not cause signs of acute systemic toxicity.	CeO_2_ NPs, citrate-stabilized	Probably low
Hematopoietic system	Radioprotective effects of ultra-small citrate-stabilized cerium oxide nanoparticles in vitro and in vivo	Popov A.L., Zaichkina S.I., Popova N.R., Rozanova O.M., Romanchenko S.P., Ivanova O.S., Smirnov A.A., Mironova E.V., Selezneva I.I., Ivanov V.K.	2016	10.1039/C6RA18566E	in vivo	SHK	Micronucleus test—48 (24 + 24) Survival—120 (60 + 60)	Intact control; control with CeO_2_ administration without irradiation	3–4 nm	8.3 nM/g body weight (100 µL 10^−6^ M) (approx. 1.43 mg/kg)	Intraperitoneally, Intravenously	Single dose	Micronucleus test: irradiation after 24 h, assessment 28 h after irradiation Survival test: daily observation for 30 days	In non-irradiated mice, administration of NPs did not cause changes in bone marrow and hematopoietic cell indices compared to intact controls. Administration of NPs before irradiation reduced cytogenetic damage to bone marrow and the level of reactive oxygen species, changed the expression of antioxidant enzymes, and increased 30-day survival of mice to 60% (i.p.) and 40% (i.v.).	CeO_2_ NPs, citrate-stabilized	Probably low
	Mitochondrial Targeted Cerium Oxide Nanoclusters for Radiation Protection and Promoting Hematopoiesis	Yang L., Ran H., Yin Y., Liu J., Lu B., Ran X., Luo S., Wang W., Yang Z., Li R.	2024	10.2147/IJN.S459607	in vivo	BALB/c	395 (255 + 140)	Intact control; control with CeO_2_ administration without irradiation	2 nm, hydrodynamic size 30 nm	10 mg/kg for radiobiological samples, 4, 6, 8, 10, 12, 14 mg/kg for toxicological samples	Intravenously	Single dose (12 h before irradiation)	Survival—30 days; blood—1, 3, 6, 9, 14, 21 days; CFU-S—8 days; CM—7 days; biochemistry and histology—3 days	Administration of TPP-PCNLs 12 h before irradiation increased mouse survival, bone marrow and spleen recovery, and reduced oxidative stress and tissue damage. In healthy non-irradiated mice, administration of NPs did not cause weight loss, changes in peripheral blood parameters, liver biochemistry, or pathological changes in bone marrow, liver, spleen, intestine, kidney, or heart.	Albumin CeO_2_ with PEG and triphenylphosphine (TPP-PCNLs)	Definitely low
Reproductive system	SF-1 mediates reproductive toxicity induced by Cerium oxide nanoparticles in male mice	Qin F, Shen T, Li J, Qian J, Zhang J, Zhou G, Tong J.	2019	10.1186/s12951-019-0474-2	in vivo	C57BL/6J	48 (36 + 12)	Yes, receiving 5% sodium carboxymethylcellulose	27.62 ± 3.01 nm	10 mg/kg 20 mg/kg 40 mg/kg	Orally	Every day for 32 days	32 days	Long-term oral administration of NPs in doses greater than 20 mg/kg disrupts the reproductive function of males, reducing the number and motility of spermatozoa, disrupting DNA integrity and testosterone synthesis, and reducing the expression of the transcription regulatory factor SF-1.	CeO_2_ NPs	Probably low
	Engineered nanoceria cytoprotection in vivo: mitigation of reactive oxygen species and double-stranded DNA breakage due to radiation exposure	Das S, Neal CJ, Ortiz J, Seal S.	2018	10.1039/c8nr04640a	in vivo	C57BL/6J	35 (30 + 5)	Yes, without nanoceria administration	5–8 nm, hydrodynamic size 10 nm	100 µL of solution with a concentration of 100 nM or 100 µM	Intravenously	Once a week for 28 days (4 injections)	28 days	Administration of NPs reduces radiation-induced damage to spermatogenic tissue in mice at radiation doses up to 5 Gy, without signs of systemic toxicity.	CeO_2_ NPs	Probably low
	Cerium oxide nanoparticle elicits oxidative stress, endocrine imbalance and lowers sperm characteristics in testes of balb/c mice	O. A. Adebayo, O. Akinloye, O. A. Adaramoye	2017	10.1111/and.12920	in vivo	BALB/C	20 (15 + 5)	Yes, without impact	<10 nm	100 µg/kg 200 µg/kg 300 μg/kg	Intraperitoneally,	3 times a week for 5 weeks	36 days	Repeated intraperitoneal administration of NPs caused a decrease in sperm motility, their morphological changes, hormonal disturbances, oxidative stress, inflammation and histopathological damage to the testicles.	PAA-CeO_2_ NPs	Probably high
	Embryo-Protective Effects of Cerium Oxide Nanoparticles against Gestational Diabetes in Mice.	Vafaei-Pour Z, Shokrzadeh M, Jahani M, Shaki F.	2018	PMID: 30127819	in vivo	Swiss	30 (20 + 10)	Yes, healthy pregnant mice without impact	No information	60 mg/kg	Intraperitoneally,	Every day for 16 days of pregnancy	16 days	In the control group of pregnant women without diabetes, the introduction of NPs did not cause significant changes in the weight of the mother, embryos, glucose levels, or markers of oxidative stress; pathological changes in the embryos were absent.	CeO_2_ NPs	Probably high
Visual system	«Non-toxic retention of nanoceria in murine eyes»	Xue Cai, Sudipta Seal, James F. McGinnis	2016	PMID: 27746672	in vivo	C57BL/6J	3-6 eyes per group (7 groups)	Yes, without impact	3–5 nm	0.1 mM (17.2 ng), 0.3 mM (51.6 ng), 1 mM (172 ng), 3 mM (516 ng) 10 mM (1720 ng)	Intravitreal	Single dose	7 h, 3 days, 7 days, 15 days, 30 days	No changes in retinal structure or function, inflammatory response, vascular permeability, or cellular infiltration were detected; photoreceptor protein localization and levels were unchanged.	CeO_2_ NPs	Probably low
	«A cerium oxide loaded glycol chitosan nano-system for the treatment of dry eye disease»	Fan Yu, Min Zheng, Alice Yang Zhang, Zongchao Han	2019	10.1016/j.jconrel.2019.10.039	in vivo	C57BL/6J	36	Yes, without impact	100 nm, hydrodynamic size 184.9 ± 8.7 nm	0.1 µM 1 µM 10 µM (2 µL per eye)	Instillation (eye drops)	2 times a day, 7 days	3 days 7 days	NPs increased tear volume, tear film breakup time, decreased fluorescein staining scores, restored corneal morphology and goblet cell density, and decreased K10 expression.	CeO_2_ NPs in glycol chitosan (GCCNP)	Probably low
	«Regenerative cerium oxide nanozymes alleviate oxidative stress for efficient dry eye disease treatment»	Haoyu Zou, Haiting Wang, Baoqi Xu, Lin Liang, Liangliang Shen, Quankui Lin	2022	10.1093/rb/rbac070	in vivo	C57BL/6J	No information	Yes, healthy mice without nanoceria administration	5–10 nm, hydrodynamic size 67.02 ± 0.73 nm	5 µL/eye, concentration not specified exactly, in in vitro experiments—10 mg/mL	Instillation (eye drops)	3 times a day, 7 days	Clinical and ophthalmic assessment on days 0, 1, 3, 5, 7; histology on day 7	In a dry eye model, NPs significantly reduced corneal damage, accelerated corneal epithelial recovery, and normalized goblet cell counts, while not causing inflammatory or pathological changes in eye tissue in healthy animals.	CNP-bPEI-g-PEG (NPs modified bPEI-g-PEG)	Probably low
Cardiovascular system	Cardioprotective effects of cerium oxide nanoparticles in a transgenic murine model of cardiomyopathy	Niu J, Azfer A, Rogers LM, Wang X, Kolattukudy PE	2007	PMID: 17207782	in vivo	FVB/N, transgenic MCP-1	24 (12 + 12)	Yes, healthy mice with and without nanoceria administration	7 nm	0.15 mM (100 µL)	Intravenously	2 times a week, 2 weeks	0, 11, 15, 19 weeks—echocardiography, 19 weeks—morphological, immunohistochemical, molecular and biochemical analysis.	In healthy mice, the administration of NPs did not cause changes in echocardiographic, histological, inflammatory and biochemical parameters compared to healthy mice without administration.	CeO_2_ NPs	Definitely low
Nervous system	Custom Cerium Oxide Nanoparticles Protect Against a Free Radical Mediated Autoimmune Degenerative Disease in the Brain	Heckman K.L., et al.	2013	10.1021/nn403743b	in vivo	SJL/J	10	Yes, healthy mice with nanoceria administration	2.9 ± 0.3 nm	20 mg/kg	Intravenously	Single dose	24 h, 1, 2, 3, 4, 5 months	Cerium was detected in the brain of healthy mice with an intact BBB; the concentration decreased over time but remained measurable after 5 months.	CeO_2_ NPs, citrate/EDTA stabilized	Probably low

## Data Availability

No new data were created or analyzed in this study.

## Abbreviations

The following abbreviations are used in this manuscript:

BAL	bronchoalveolar lavage
BBB	blood–brain barrier
CA	citric acid
CNS	central nervous system
EDTA	ethylenediaminetetraacetic acid
GIT	gastrointestinal tract
ICP-MS	inductively coupled plasma mass spectrometry
MDA	malondialdehyde
NPs	cerium dioxide nanoparticles, nanoceria
OS	oxidative stress
PAA	polyacrylic acid
PBS	phosphate-buffered saline
PT	prothrombin time
ROS	reactive oxygen species
SOD	superoxide dismutase
TEM	transmission electron microscopy
TI	toxicological impact
WT	wild type
